# SARS-CoV-2 envelope protein causes acute respiratory distress syndrome (ARDS)-like pathological damages and constitutes an antiviral target

**DOI:** 10.1038/s41422-021-00519-4

**Published:** 2021-06-10

**Authors:** Bingqing Xia, Xurui Shen, Yang He, Xiaoyan Pan, Feng-Liang Liu, Yi Wang, Feipu Yang, Sui Fang, Yan Wu, Zilei Duan, Xiaoli Zuo, Zhuqing Xie, Xiangrui Jiang, Ling Xu, Hao Chi, Shuangqu Li, Qian Meng, Hu Zhou, Yubo Zhou, Xi Cheng, Xiaoming Xin, Lin Jin, Hai-Lin Zhang, Dan-Dan Yu, Ming-Hua Li, Xiao-Li Feng, Jiekai Chen, Hualiang Jiang, Gengfu Xiao, Yong-Tang Zheng, Lei-Ke Zhang, Jingshan Shen, Jia Li, Zhaobing Gao

**Affiliations:** 1 CAS Key Laboratory of Receptor Research, Stake Key Laboratory of Drug Research, Shanghai Institute of Materia Medica, Chinese Academy of Sciences, Shanghai, China; 2 University of Chinese Academy of Sciences, Beijing, China; 3 State Key Laboratory of Virology, Wuhan Institute of Virology, Center for Biosafety Mega-Science, Chinese Academy of Sciences, Wuhan, Hubei China; 4 Key Laboratory of Animal Models and Human Disease Mechanisms of the Chinese Academy of Sciences/Key Laboratory of Bioactive Peptides of Yunnan Province, KIZ-CUHK Joint Laboratory of Bioresources and Molecular Research in Common Diseases, Kunming Institute of Zoology, Chinese Academy of Sciences, Kunming, Yunnan China; 5 Shanghai University of Medicine & Health Sciences, Shanghai, China; 6 State Key Laboratory of Genetic Resources and Evolution, Kunming Institute of Zoology, Chinese Academy of Sciences, Kunming, Yunnan China; 7 Kunming National High-level Biosafety Research Center for Non-human Primates, Center for Biosafety Mega-Science, Kunming Institute of Zoology, Chinese Academy of Sciences, Kunming, Yunnan China; 8 Center for Cell Fate and Lineage (CCLA), Guangzhou Regenerative Medicine and Health Guangdong Laboratory (GRMH-GDL), Guangzhou, Guangdong China; 9 CAS Key Laboratory of Regenerative Biology, Guangdong Provincial Key Laboratory of Stem Cell and Regenerative Medicine, Guangzhou Institutes of Biomedicine and Health, Chinese Academy of Sciences, Guangzhou, Guangdong China; 10 Joint School of Life Science, Guangzhou Medical University, Guangzhou, Guangdong China; 11 Zhongshan Institute of Drug Discovery, Institution for Drug Discovery Innovation, Chinese Academy of Science, Zhongshan, Guangdong China; 12 School of Pharmacy, Fudan University, Shanghai, China

**Keywords:** Cell death, Molecular biology

## Abstract

Cytokine storm and multi-organ failure are the main causes of SARS-CoV-2-related death. However, the origin of excessive damages caused by SARS-CoV-2 remains largely unknown. Here we show that the SARS-CoV-2 envelope (2-E) protein alone is able to cause acute respiratory distress syndrome (ARDS)-like damages in vitro and in vivo. 2-E proteins were found to form a type of pH-sensitive cation channels in bilayer lipid membranes. As observed in SARS-CoV-2-infected cells, heterologous expression of 2-E channels induced rapid cell death in various susceptible cell types and robust secretion of cytokines and chemokines in macrophages. Intravenous administration of purified 2-E protein into mice caused ARDS-like pathological damages in lung and spleen. A dominant negative mutation lowering 2-E channel activity attenuated cell death and SARS-CoV-2 production. Newly identified channel inhibitors exhibited potent anti-SARS-CoV-2 activity and excellent cell protective activity in vitro and these activities were positively correlated with inhibition of 2-E channel. Importantly, prophylactic and therapeutic administration of the channel inhibitor effectively reduced both the viral load and secretion of inflammation cytokines in lungs of SARS-CoV-2-infected transgenic mice expressing human angiotensin-converting enzyme 2 (hACE-2). Our study supports that 2-E is a promising drug target against SARS-CoV-2.

## Introduction

Severe acute respiratory syndrome coronavirus 2 (SARS-CoV-2) has caused a worldwide pandemic. Cytokine storm and consequent acute respiratory distress syndrome (ARDS) characterized by dysfunctional immune responses and severe pulmonary injury are the main causes of SARS-CoV-2-related death.^1–3^ The excessive responsive secretion of inflammatory cytokines (IL-1β, IL-6, IL-10, TNF-α, etc.) and chemokines (CXCL10, CXCL9, CCL2, CCL3, CCL5, etc.) is readily followed by the immune system “attacking” various tissues, which causes ARDS.^4^ After the inflammatory outburst, immune system in severe COVID-19 patients may be programmed to an immunosuppression condition, which leads to death due to the collapse of the whole immune system.^5^ Intensive studies on host–pathogen interactions have partially unveiled the immunopathogenesis of COVID-19.^6–8^ Similar to two other beta-coronaviruses, severe acute respiratory syndrome coronavirus (SARS-CoV) and Middle East respiratory syndrome coronavirus (MERS-CoV), which could cause fatal pneumonia,^9–11^ SARS-CoV-2 can also induce secretion of inflammatory cytokines and cell death by releasing pathogen-associated molecular patterns (PAMPs), such as viral RNA and damage-associated molecular patterns (DAMPs), including Adenosine triphosphate (ATP) and DeoxyriboNucleic Acid (DNA). It is also very likely that SARS-CoV-2 may antagonize interferon responses in various interferon signaling pathways according to studies on SARS-CoV.^2,12–14^ However, in addition to the dysfunctional defensive responses of host cells and the antagonism of the interferon signaling pathways by the virus, it remains unknown whether the SARS-CoV-2 itself possesses an offensive virulence factor that can induce cytokine storm and kill targeted cells in a direct and rapid manner.

SARS-CoV-2 is an enveloped, positive-sense, single-stranded RNA beta-coronavirus. It contains four structural proteins, Spike (S), Membrane (M), Nucleocapsid (N) and Envelope (E) protein, all of which are needed for the formation of mature virus particles.^15^ The S protein of SARS-CoV-2 is a glycoprotein on the virus surface and consists of two domains S1 (14–667 aa) and S2 (668–1255 aa). S1 recognizes and binds the human angiotensin-converting enzyme 2 (hACE-2) of the host surface, and S2 forms a six-helix conformation, mediating the fusion of the viral envelope with the host cell plasma membrane.^16–18^ Because of its essential role for initial invasion of host cells, S protein has become a main focus of antiviral target for vaccine development and drug design.^16^ M protein (222 aa) is a highly conserved non-glycosylated membrane protein, which is mainly located on the inner surface of the viral envelope and constitutes a scaffold connecting the viral inner membrane with the N protein. In the early stage of viral infection, M protein is accumulated in the host cell nucleus and inhibits the transcription and translation of host cell genes, allowing sufficient time for the viral genome replication, transcription and translation. While in the late stage of infection, M protein enters the cytoplasm and assists the assemble and release of progeny virus particles.^19^ N protein (419 aa) is located at the center of the SARS-CoV-2 virus particles and interacts with virus RNA, playing a key role in packaging viral RNA genome during virus assembly.^20^ Among the four major structural proteins, SARS-CoV-2 envelope protein (2-E) is the smallest one with only 75 aa and is assumed to be a transmembrane protein.^15^ Its C-terminal domain may interact with host cell intracellular protein Caenorhabditis elegans lin-7 protein 1 (PALS1), which is critical for the maintenance of epithelial polarity in mammals.^21^ More knowledge on envelope (E) protein has been limited from a few studies on the highly conserved E protein of SARS-CoV that emerged in 2003.^22^ E protein contains a hydrophilic amino terminal domain (NTD), a hydrophobic transmembrane domain (TMD), and a long and hydrophilic carboxyl terminal domain (CTD).^22^ It has been reported that the E protein of SARS-CoV may form pores with ion channel activity.^23–25^ The expression of the E protein during virus replication was thought to be essential for SARS-CoV assembly and budding.^26^ A recent study argued that the SARS-CoV-E protein did not participate in virus production but was an independent virulence factor.^27^ It was thought that the SARS-CoV-E protein may also act as a proapoptotic protein inducing apoptosis through activating endoplasmic reticulum (ER) stress or interacting with other proteins.^22,28^ To date, however, the drug ability of E proteins of coronavirus has never been validated.

Here, by using a combination of biochemical, electrophysiology approaches and animal models, we showed that the 2-E protein formed a cation channel, which was lethal to host cells and even healthy surrounding cells, and inhibition of the 2-E channel is a promising antiviral strategy.

## Results

### SARS-CoV-2-E forms a cation channel

**Fig. 1:**
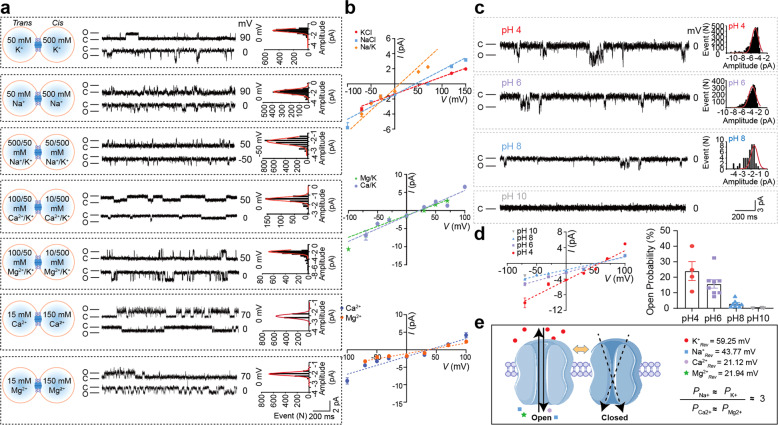
SARS-CoV-2-E forms a cation channel. **a** Single-channel current recording of 2-E after reconstitution in lipid bilayers with PC/PS = 3:2 lipids at the indicated potentials and solutions. Protein (5–50 ng/mL) was added to the *cis* side. All-point current histograms for the left trace (0, 0, –50, 0, 0, 0, 0 mV) (“C” means Closed; “O” means Open). **b** *I*-*V* curves of 2-E proteins in different solutions as the left panels (*n* ≥ 3). **c** Representative single-channel current recordings of 2-E in the presence of various pH in 0 mV. Right showed the amplitude histograms by Gaussian fit. The currents were recorded in asymmetric 50:500 mM KCl solutions (*trans:cis*) and the pH of both *cis* and *trans* sides were changed at the same time. **d** *I*–*V* plot (Left) and open probability (Right) were shown in different pH conditions (*n* ≥ 3). **e** Schematic diagram of 2-E channel open and closed states. The reverse potential and permeability of each ion was shown. All error bars are SEM.

Potential electric signals mediated by 2-E proteins were assessed using the planar lipid bilayer recording technique (Supplementary information, Fig. S1a, b). Typical single-channel currents were captured when the protein was reconstituted in asymmetric 50:500 mM KCl solutions and 3:2 PC/PS lipid membranes at applied potentials of –100 mV to +150 mV (Fig. 1a, b). The measured reversal potential (59.25 mV) was very close to the theoretical equilibrium potential of K^+^, indicating that the 2-E channels were permeable to K^+^ but not to Cl^–^. By examining the reversal potentials in solutions containing varied concentrations of K^+^, Na^+^ and Cl^–^, we found the 2-E channels were permeable to Na^+^ in the presence of K^+^ and Cl^-^ (Fig. 1a, b). Interestingly, the 2-E channels were also permeable to divalent Ca^2+^ and Mg^2+^. However, the permeability to divalent Ca^2+^ and Mg^2+^ was lower than that to monovalent ions (Fig. 1a, b and e). The permeability rank ((*P*_Na_^+^ ≈ *P*_K_^+^)/(*P*_Ca_^2+^ ≈ *P*_Mg_^2+^) ≈ 3.0) suggested that 2-E channel possessed a distinct selectivity pattern from classic voltage-dependent channels. The pH value on the lumen side of organelles may change after coronavirus infection.^26^ We found that both the amplitude and open probability gradually increased when the pH decreased in independent experiments. At pH 10, the 2-E channel almost completely closed under 0 mV (Fig. 1c, d; Supplementary information, Fig. S1c, d). Consistently, a most recent resolved transmembrane domain structure validated that 2-E indeed forms a homopentameric channel and its N- and C-terminal halves are sensitive to pH change.^29^ Collectively, our data support that 2-E forms a type of pH-sensitive cation channel which is consistent with the roles of homologous E proteins in the life cycle of SARS-CoV.^27^

### SARS-CoV-2-E induces cell death in vitro

**Fig. 2:**
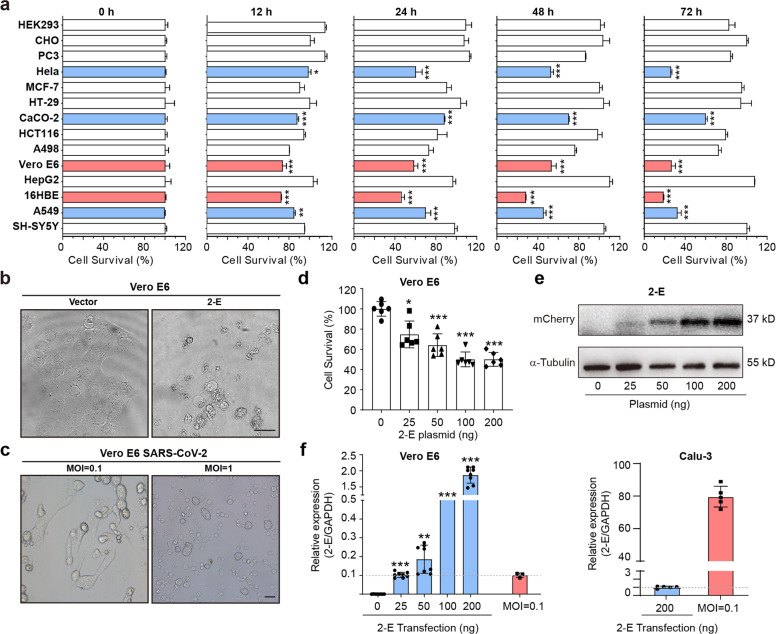
SARS-CoV-2-E expression induces cell death in vitro as SARS-CoV-2 did. **a** Cell viability of 14 cell lines at the indicated time after transfection with 2-E plasmids. **b** Microscopy images of 2-E expression in Vero E6 cells during cell death (bar, 10 μm). **c** Images of Vero E6 cells infected with SARS-CoV-2 virus (bar, 25 μm). Vero E6 and Calu-3 cells were transfected with 2-E-mCherry plasmid at indicated concentration. **d** Survival viability of Vero E6 cells under different transfection levels. **e** Protein expression levels of 2-E in Vero E6 cells after transfection. **f** Expression of 2-E present in 2-E-transfected and SARS-CoV-2-infected Vero E6 (left) and Calu-3 (right) cells (MOI = 0.1). All data are representative of three independent experiments. *P < 0.05; **P < 0.01; ***P < 0.001; unpaired Student’s *t-*test. All error bars are SEM.

It has been reported that the executers of pyroptosis (GSDMD) and necroptosis (p-MLKL) destroy the membrane integrity by either forming pores or channels.^30,31^ The influences of 2-E channels on host cells were evaluated in 14 cell lines and the survival rate of each cell line was measured at 12, 24, 48 and 72 h after transient transfection. Among them, Vero E6 (African green monkey kidney), 16HBE (human bronchial epithelium), A549 (human alveolar basal epithelium), HeLa (human cervical cancer) and CaCO-2 (human intestinal carcinoma) cells gradually died while the rest remained in a healthy state (Fig. 2a). A relatively high expression level of 2-E was observed in the susceptible cell lines, while the rest of the cell lines displayed no expression (Supplementary information, Fig. S2). Vero E6 cells were selected for further examination. After transfection with 2-E plasmids, the initial smooth surface of the dying cell expressing 2-E started to swell, eventually exploded and released cellular contents (Fig. 2b). Meanwhile, flow cytometry confirmed that the cells expressing 2-E proceeded to the Annexin V and Propidium iodide (PI) double-positive stage (Supplementary information, Fig. S3). The double-positive results represent phosphatidylserine (PS) exposure and loss of plasma membrane integrity, which are hallmarks of cell death.^32,33^ It was worth noting, SARS-CoV-2 infection caused severe Vero E6 cell death, which is also characterized by bubbling (Fig. 2c). However, among the tested biomarkers for necroptosis and pyroptosis, levels of neither phosphorylated mixed lineage kinase domain-like protein (p-MLKL, necroptosis) nor cleaved gasdermin D (GSDMD, pyroptosis) were increased (Supplementary information, Fig. S4). These data suggest that 2-E induce pyroptosis-like cell death in an atypical manner. Next, whether 2-E protein could induce cell death at physiological concentrations was further evaluated. Due to the lack of antibody for 2-E protein, we compared the cellular mRNA levels of 2-E with Quantitative real-time PCR (qRT-PCR) in both heterologous expression system (transfection of 2-E plasmids 25–200 ng per well, approximately 3–25 ng/10^3^ cell) and SARS-CoV-2 viral (MOI = 0.1) infection system (Fig. 2d–f). For Vero E6 cells, the mRNA level of 2-E in SARS-CoV-2-infected cells was comparable to that in cells transiently transfected with 25 ng plasmid, our lowest DNA level tested for transfection. Importantly, the mRNA level of 2-E in Calu-3 cells infected with SARS-CoV-2 was 80-fold higher than cells transiently transfected with the highest amount of 2-E plasmids DNA level (200 ng), suggesting that the 2-E effects may be more pronounced in SARS-CoV-2 viral infection in vitro and in vivo than the cells transfected with 2-E expression plasmids. Altogether, these data suggest that the 2-E-induced cell death could play critical roles in pathogenesis.

All seven human coronaviruses, including HCoV-229E, HCoV-NL63, HCoV-OC43, SARS-CoV, HCoV-HKU1, MERS-CoV and SARS-CoV-2, could cause respiratory system damages. According to their transmission capacity, infected population, disease severity and fatality rate, human coronaviruses can be divided into low pathogenic and highly pathogenic coronaviruses.^34^ The E proteins of these different coronaviruses are highly conserved with around 40% sequence homology. In particular, the homology between the E proteins of SARS-CoV and SARS-CoV-2, two highly pathogenic coronaviruses, is as high as 95% (Supplementary information, Fig. S5a). Consistent with the previous reports, the E protein of SARS-CoV indeed forms cation-permeable channels (Supplementary information, Fig. S5b).^24^ We found that it caused similar cell death to 2-E did when heterogeneously expressed in Vero E6 cells (Supplementary information, Fig. S5c). Interestingly, the E protein of HCoV-OC43, a low pathogenic coronavirus, also displayed channel activity. However, the expression level of HCoV-OC43-E needed to be much higher to be lethal to cells (Supplementary information, Figs. S5d, e). Notably, multiple additional factors, such as the invasion ability, the degree of virus replication and virus transport efficiency, have been also found to contribute to the pathogenesis of coronaviruses.^34–36^ How the 2-E channels affect the pathogenesis of SARS-CoV-2 infection is worth investigating.

### SARS-CoV-2-E provokes robust immune responses in vitro and in vivo

**Fig. 3:**
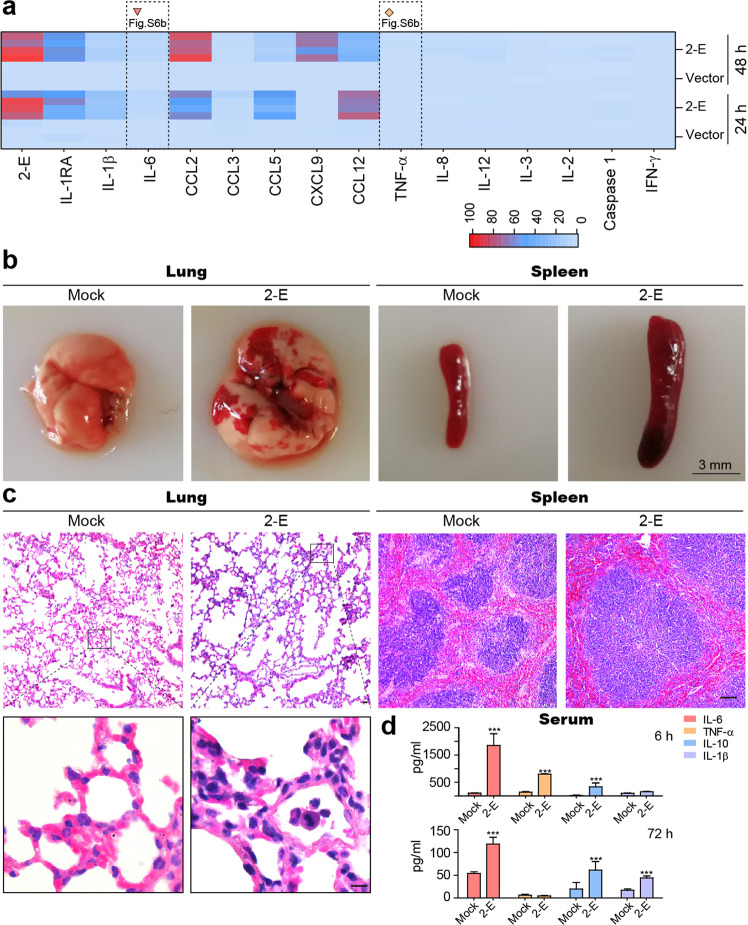
SARS-CoV-2-E provokes robust immune responses in vitro and in vivo. **a** Differentially expressed genes associated with the defense response to transfection of 2-E were summarized in a heat-map. The color code presents a linear scale. Red triangle and yellow rhombus, time courses of the levels of IL-6 and TNF-α after transfection with 2-E plasmids (Supplementary information, Fig. S6b). **b**, **c** Gross pathology (**b**) and histopathology (**c**) of lungs (left) and spleens (right) from control mice (Mock, TBS) and model mice (2-E, 2-E proteins) (bar, 10 μm). **d** Serum cytokine levels at 6 h and 72 h after treatment. *P < 0.05; **P < 0.01; ***P < 0.001; unpaired Student’s *t*-test. All error bars are SEM.

Previous studies have shown that the SARS-CoV-E protein is associated with macrophage infiltration and inflammatory cytokine production.^27,37^ Three methods were used to explore the 2-E-induced pathological processes in macrophages (Supplementary information, Fig. S6a). First, we transfected a mouse macrophage cell line (RAW264.7) with 2-E plasmids for 24 h and 48 h. As shown in Fig. 3a, expression levels of cytokines like TNF-α and IL-6, and chemokines like CXCL9 and CCL12 were significantly upregulated in cells and supernatants after transfection, as previously observed in COVID-19 patients^12^ (Fig. 3a; Supplementary information, Fig. S6b, c and Table S1). Second, we treated macrophages with purified 2-E proteins and obtained similar enhanced cytokine levels (Supplementary information, Fig. S6d). Given that virus infection is related to innate and adaptive immune responses, it is logical to speculate that host cell damage by 2-E would lead to a secondary inflammatory cascade. To test this hypothesis, we transfected Vero E6 with 2-E plasmids, and 24 h later, the culture supernatant was collected and used to treat RAW264.7 cells. As expected, the supernatant of impaired cells promoted cytokine expression as well (Supplementary information, Fig. S6e). We then intravenously injected C57BL/6 mice with purified 2-E proteins for 6 h and 72 h. Surprisingly, we observed severe foci of pulmonary consolidation in the lung and spleen edema at 72 h (Fig. 3b), but not in mock and bovine serum albumin (BSA) groups (Supplementary information, Fig. S7). Hematoxylin-eosin (H&E) staining of lung tissues showed marked inflammatory cell infiltration, edema, pulmonary interstitial hyperemia, hemorrhage, and alveolar collapse. These observations were similar to the COVID-19 patients’ lungs, in which the first and most severe lesion signs appeared^3^ (Fig. 3b). SARS-CoV-2 infection may trigger an overwhelming inflammatory response, which leads to injuries in multiple organs.^13,38,39^ We used qRT-PCR and enzyme linked immunosorbent assay (ELISA) to characterize the pathological features. The expression of cytokines and chemokines increased dramatically in vivo, along with the notably upregulated inflammatory markers, which was also correlated with the previously reported cytokine storm in patients (Fig. 3c; Supplementary information, Fig. S8). Our data suggest that 2-E protein alone is able to drive pulmonary congestion and provoke robust immune responses in vitro and in vivo.

### Dominant negative mutation Thr11Ala (2-E^T11A^) weakens SARS-CoV-2 replication and virulence by impairing 2-E channel function

**Fig. 4:**
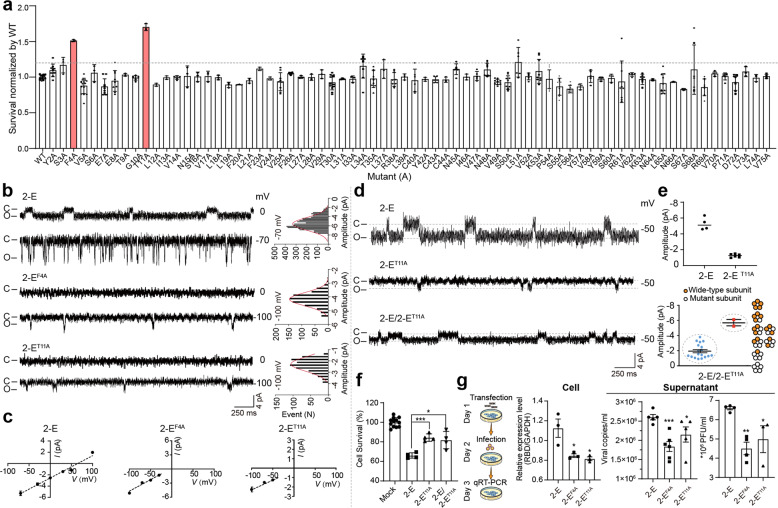
Dominant negative mutation Thr11Ala (2-E^T11A^) weakens SARS-CoV-2 replication and virulence by impairing 2-E channel function. **a** Survival level of each mutant. Each residue of 2-E protein was substituted with alanine (A) and the influences of each mutation on cell death induction was examined via Cell Counting Kit-8 (CCK-8). **b** Representative traces for 2-E, 2-E^F4A^ and 2-E^T11A^ in lipid bilayers with PC/PS = 3:2 lipids at the indicated potentials. **c** *I*–*V* curves of 2-E, 2-E^F4A^ and 2-E^T11A^ (*n* ≥ 3). **d** Representative traces for 2-E alone, 2-E^T11A^ alone and mixture of 2-E/2-E^T11A^ (1:1). **e** Current amplitude of 2-E and 2-E^T11A^ (up); current amplitude of mixture of 2-E/2-E^T11A^ (1:1) and the cartoon illustrated the model of pentamer (down). **f** Survival level for Vero E6 cells after transfection with plasmids as indicated. **g** Flow chart of the experiment (left), and the virus copies in cells and supernatant determined by qRT-PCR assay (right). MOI of SARS-CoV-2 for infection was 0.01. Experiments were independently performed twice and similar results were obtained. One set of reprehensive data is shown here (*n* ≥ 3) (**f**, **g**). *P < 0.05; **P < 0.01; ***P < 0.001; unpaired Student’s *t*-test. All error bars are SEM.

The functional implications of the 2-E channels were investigated using mutants obtained from the alanine-scanning strategy. Each residue was substituted with alanine (A) and the influences of each mutation on cell death induction was examined individually. Among the total 70 mutants, we found that two mutants Phe4Ala (2-E^F4A^) and Thr11Ala (2-E^T11A^) significantly alleviated cell death compared with wild-type (WT, 2-E) and other mutant channels (Fig. 4a). Immunofluorescence images and western blot results suggested that the two mutations did not alter the transfection efficiency and expression levels (Supplementary information, Fig. S9). How these two mutants affect cytokine release and the pathological phenotype in vivo was then evaluated. We found that, in comparison with WT channels, heterogeneous expression of the mutants caused less cytokine release in RAW264.7 cells and inoculating the mutant proteins to mouse caused weaker lung pathological phenotype in vivo (Supplementary information, Figs. S10–12). Then we purified the mutant proteins (2-E^F4A^ and 2-E^T11A^) and performed single-channel recording using bilayer lipids system. Consistent with their inability to induce cell death, both mutants failed to induce typical single-channel currents under stimulation potential higher than 0 mV. Only with stimulation potential at or below –50 mV, a much diminished current could be recorded from mutant channels (Fig. 4b, c). The mutant 2-E^T11A^ was selected for further investigation. We thoroughly mixed 2-E^WT^ and 2-E^T11A^ proteins at 1:1 ratio and recorded the channel currents after random combination. With 20 independent detection experiments, we found that the recorded single-channel currents could be divided into two distinct groups according to current amplitudes. One group with smaller amplitudes ranged from –0.8 to –3.3 pA. The proportion was 18/20 (more n-numbers). The other group with larger amplitude, approximated –5.8 pA. The proportion was 2/20 (less n-numbers). The average amplitude of the smaller group was about –1.69 pA close to 2-E^T11A^ alone (–1.6 pA), whereas that of larger group was –5.8 pA resembling 2-E^WT^ alone (–5.7 pA) (Supplementary information, Fig. S13). These data suggested that the mutant 2-E^T11A^ and 2-E^WT^ could assemble together and form heteromultimer with various stoichiometry and the mutation 2-E^T11A^ exerted a dominant negative effect on 2-E channel currents (Fig. 4d, e). The probability ratio of events with larger amplitudes vs smaller amplitudes was 1:10. A recent structure study validated that the 2-E transmembrane domain formed a homopentamer as the E protein of SARS-CoV does,^29^ and we suggest that one or more mutant subunits in a pentameric channel would dominantly impair the channels’ function.

In order to evaluate the functional implication of 2-E^T11A^, we first tested whether the mutation 2-E^T11A^ affects the capability of 2-E-induced cell death. Vero E6 cells were transfected with mixture of 2-E^WT^/2-E^T11A^ (1:1), 2-E^WT^ alone and 2-E^T11A^ alone, and the cell survival ratios were examined, respectively. Consistently, 2-E^WT^/2-E^T11A^ or 2-E^T11A^ transfected cells showed much higher survival rate compared with 2-E^WT^ (Fig. 4f). Interestingly, the protein expression levels of 2-E^WT^/2-E^T11A^ and 2-E^T11A^ were actually higher than that of 2-E^WT^, supporting that the dominant negative mutation indeed mitigated the cellular lethality by decreasing 2-E channel activity rather than lowering protein expression level (Supplementary information, Fig. S9c).

The next question is what are the roles of protein 2-E, as an ion channel, played in SARS-CoV-2 life cycle, and whether the 2-E channel activity could have physiological implications and therapeutic values. We designed the following experiments to address these questions. At day 1, Vero E6 cells were transfected with 2-E^WT^ or 2-E^T11A^ plasmids, and then infected with SARS-CoV-2 virus at day 2. As determined by qRT-PCR, pre-expression of mutant 2-E^T11A^ significantly attenuated viral loadings and titers compared with 2-E^WT^ indeed in both cells and supernatants (Fig. 4g). It was logic to deduce that some exogenous 2-E^T11A^ subunits would co-assemble with the WT subunits from the virus and form heteromultimers with impaired channel activity due to the dominant negative effects of the mutation 2-E^T11A^, which then greatly hampered the SARS-CoV-2 virus production. Altogether, these results support that 2-E functions as an ion channel physiologically in SARS-CoV-2 life cycle.

### Newly identified channel inhibitors exhibit protective effects against 2-E-induced damage and anti-SARS-CoV-2 activity in vitro

**Fig. 5:**
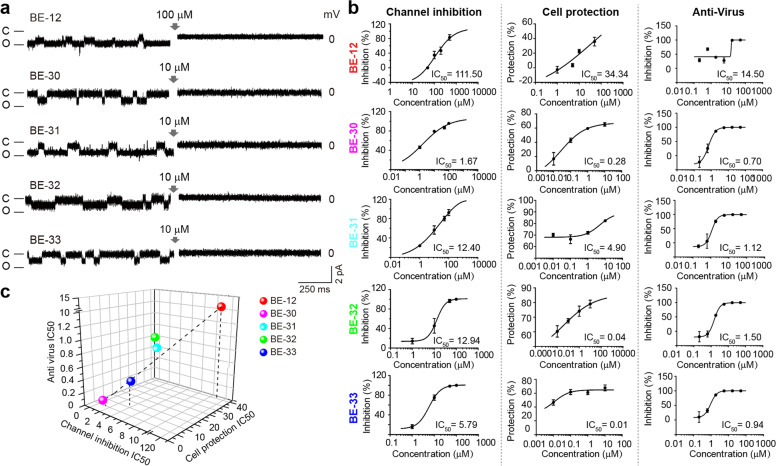
Newly identified channel inhibitors exhibit protective effects against 2-E-induced damage and anti-SARS-CoV-2 activity in vitro. **a** Representative single-channel traces after exposure to the indicated compounds at indicated concentrations. Once ion channel conductance was detected, compounds were added to the *trans* chamber while stirring to facilitate binding of the compound to the channel. The gray arrow indicates the application of compounds (*n* ≥ 3). **b** Dose–response curves of the indicated inhibitors on channel activity (left), 2-E-induced cell death (middle) and SARS-CoV-2 infection in Vero E6 cells (right). **c** The correlation among the IC_50_s for channel inhibition, cell protection and antiviral activity.

We sought to identify inhibitors for 2-E channels using the planar lipid bilayer recording system. Tetraethylammonium (TEA, 5 mM), Tetrodotoxin (TTX, 100 μM) and Nifedipine (100 μM), three classic inhibitors of potassium, sodium and calcium channels, respectively, showed negligible effects on 2-E channels. Although 5-(N, N-hexamethylene)-amiloride (HMA) and amantadine, two reported inhibitors of proton pumps or channels, exhibited inhibitory activity on 2-E channels at 50 μM or greater, further investigation into these compounds was hindered by their strong cytotoxicity (Supplementary information, Fig. S14). BE-12 (Berbamine), a type of bisbenzylisoquinoline alkaloid isolated from traditional Chinese herbal medicines such as *Berberis amurensis*, drew our attention after screening an in-house compound collection. Although BE-12 was a weak inhibitor of 2-E channels, with an IC_50_ of 111.50 μM, it exhibited minor cytotoxicity, which allowed us to further test its cell protection activity against 2-E-induced cell death and anti-SARS-CoV-2 activity using Vero E6 cells. Encouraged by the promising cell protection and antiviral activity of BE-12, four more channel inhibitors (BE-30~33) were designed, synthesized and evaluated individually (Supplementary information, Fig. S15). Among them, BE-33 exhibited exceptional antiviral activity against SARS-CoV-2 infection, with an IC_50_ of 0.94 μM and negligible cytotoxicity (Fig. 5a, b; Table 1). The binding of BE-33 to 2-E channels was validated using surface plasmon resonance (SPR) (Supplementary information, Fig. S16). It is noteworthy that the channel inhibition activity, the cell protection activity against 2-E-induced cell death and the antiviral activity in vitro of this class of compounds are positively correlated with each other (Fig. 5c). Collectively, these results indicate that inhibition of 2-E channel activity is likely to be a potential therapeutic strategy against SARS-CoV-2 infection.

**Table 1:** Activity of newly identified 2-E channel inhibitors in vitro.

Name	Structure	IC_50_ (μM)				Selection index (SI)
		Channel inhibition^a^	Cell protection^b^	Anti-virus^c^	Cytotoxicity^d^	
**BE-12 (Berbamine)**		**111.50**	**34.34**	**14.50**	**29.94**	**2.06**
**BE-30**		**1.67**	**0.28**	**0.70**	**25.59**	**36.56**
**BE-31**		**12.40**	**4.90**	**1.12**	**70.55**	**62.99**
**BE-32**		**12.94**	**0.04**	**1.50**	**172.20**	**114.80**
**BE-33**		**5.79**	**0.01**	**0.94**	**31.46**	**33.47**

All data are representative of three independent experiments.

^a^The planar lipid bilayer recording technique was used to assess the inhibition of the indicated compounds with a serial concentration of 10, 50, 100, 200, 500 μM for BE-12 and 1, 10, 50, 100 μM for BE-30, BE-31, BE-32, and BE-33. The concentration of the tested compound that results in a half-maximal decrease in the open probability is defined as IC_50_.

^b^At 24 h post transfection, Vero E6 cell survival rate was determined by using CCK-8 assays. The compound concentrations: 0.01, 0.1, 1 and 10 μM.

^c^At 24 h post infection, viral RNA copy in the cell supernatant was quantified by qRT-PCR. The compound concentrations: 0.21, 0.62, 1.85, 5.56, 16.7 and 50 μM.

^d^The cytotoxicity of these compounds in Vero E6 cells was also determined by using CCK-8 assays. The compound concentrations: 0.08, 0.23, 0.69, 2.1, 6.2, 18.5, 55.6, 167 and 500 μM.

### Prophylactic and therapeutic benefits of 2-E channel inhibitor on SARS-CoV-2-infected hACE-2 mouse model

**Fig. 6:**
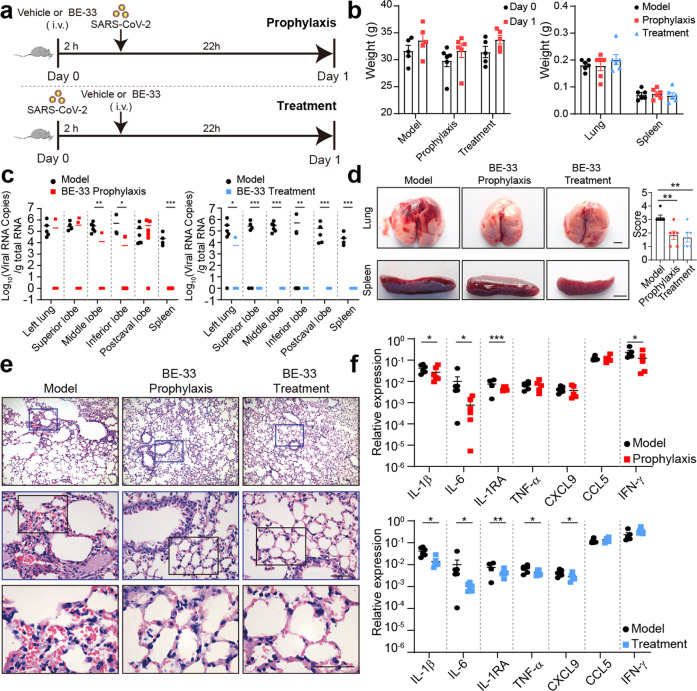
2-E channel inhibitor protects mice against SARS-CoV-2 infection and limits inflammation in the lung. **a** Scheme of experimental parameters. The dose of SARS-CoV-2 was 2 × 10^6^ TCID_50_ per mouse. **b** Body (Left), lung and spleen (Right) weight after infection with SARS-CoV-2 and injection with BE-33. **c** Viral RNA loading of different parts of mouse lungs after infection with SARS-CoV-2 and injection with BE-33. **d** Lesions in the lungs and spleens. A view of the ventral lungs and spleens of an infected animal obtained at necropsy post infection (left) (Scale bars, 2.5 mm). The pathology score was on the right. **e** H&E staining of lung sections (Scale bars, 50 μm). Each image is representative of a group of 3 mice. **f** Left lungs of each group were evaluated for cytokine and chemokine expression by qRT-PCR assay. *n* > 5 for each group, and mice were killed at different time as indicated. *P < 0.05; **P < 0.01; ***P < 0.001; Student’s *t-*test. All error bars are SEM.

The in vivo antiviral activity of 2-E channel inhibitor was evaluated in transgenic mice expressing hACE-2, a common animal model of SARS-CoV-2 infection.^38,40–43^ We determined the in vivo efficacy of BE-33 via two modes of administration. Either, the ACE-2 humanized mice (hACE-2 mice) were subjected to prophylactic dosing with 5 mg/kg BE-33 or vehicle as a control 2 h prior to infection with SARS-CoV-2 (2 × 10^6^ TCID_50_). Or, the transgenic hACE-2 mice were treated with 5 mg/kg BE-33 2 h after infection with SARS-CoV-2 (2 × 10^6^ TCID_50_) (Fig. 6a). We monitored the pathological condition of mice one day after infection. At the end of the study, all mice were alive and sacrificed to collect tissues. Body, lung and spleen weight were detected, and no obvious changes were observed (Fig. 6b). The viral replication of SARS-CoV-2 was measured by qRT-PCR assay as previous studies.^4,44^ For mice receiving BE-33, we observed a trend toward decreased levels of viral RNA in the lung for both therapeutic or preventive administration in comparison with levels seen in the control mice. Especially in the therapeutic group, the viral copies of all lung lobes dropped below the detection limit (Fig. 6c). Necropsy was then performed and the postmortem examinations showed a severe foci of pulmonary, a variable degree of consolidation, edema, hemorrhage and necrosis throughout the lower respiratory tract, indicating the interstitial pneumonia in control group, and BE-33 could relieve these symptoms in experimental group (Fig. 6d, upper panel). The edema of spleen was significantly alleviated after BE-33 administration, especially in therapeutic group (Fig. 6d, bottom panel). Upon histopathological analysis, the mouse lung pathology caused by SARS-CoV-2 infection resembles that seen in severe cases of human SARS-CoV-2 infection, with diffuse alveolar damage, pulmonary edema, hyaline membrane formation, and infiltration of lymphocytes into the alveolar septa, whereas there were only few observed eosins in the lungs and minor damage of mouse lung in BE-33-treated group (Fig. 6e). To assess whether BE-33 can alleviate virus-induced inflammation, we measured the mRNA levels of inflammatory cytokines and chemokines in the left lung (Fig. 6f). Mice treated with BE-33 had significantly lower levels of inflammatory cytokines and partial chemokines than infected mice. Specifically, IL-6, IL-1RA and IL-1β were decreased during infection in both therapeutic and preventive groups. The in vivo antiviral activity of the channel inhibitor BE-33 supports 2-E channel as a promising drug target.

To further assess the drugability of BE-33, pharmacokinetics (PK) properties and toxicity in vivo of the inhibitor were evaluated. As shown in Table 2, BE-33 given to mice orally (10 mg/kg) and intravenously (2.5 mg/kg) displayed a half-life (T_1/2_) of 4.45 and 5.67 h, respectively, and a high maximal plasma concentration (C_max_ = 497 ± 80 ng/mL), a moderate clearance (CL = 31.1 ± 7.9 mL/min/kg) and a good oral bioavailability of 51% were observed (Table 2). In addition, BE-33 was enriched in the lungs at very high concentrations after treatment (1 × 10^5^ ng/g), which is 150 times than its antiviral IC_50_ in vitro. In vivo toxicity study of BE-33 was carried out in mice (Table 3). The dose range toxicity of BE-33 was studied for 5 days at dosing levels of 5, 10, and 50 mg/kg in C57BL/6 mice. All animals received once-daily dosing by tail vein injection and were clinically observed at least once a day. No obvious toxicity was observed in either 5 mg/kg or 10 mg/kg group. No significant changes in body weight were observed with up to 50 mg/kg of BE-33 compared with the control group (Table 3). These data suggest that BE-33 is a promising antiviral drug candidate against SARS-CoV-2 infection.

**Table 2:** Preliminary pharmacokinetic (PK) evaluation of compound BE-33 in C57BL/6 mice^a^.

Dose	Route	T_1/2_ (h)	T_max_ (h)	C_max_ (ng/mL)	AUC_last_ (h*ng/mL)	AUC_INF_obs_ (h*ng/mL)	CL (mL/min/kg)	MRT_INF_obs_ (h)	Vss__obs_ (mL/kg)	F (%)
10 mg/kg	po	4.45 ± 0.67	0.25 ± 0.00	497 ± 80	2699 ± 791	2776 ± 806	–	6.67 ± 0.47	–	51
2.5 mg/kg	iv	5.67 ± 1.09	–	–	1324 ± 330	1405 ± 393	31.10 ± 7.90	7.53 ± 1.90	13535 ± 1699	–

*T*_*1/2*_ half-life, *T*_*max*_ time to maximum plasma concentration, *C*_*max*_ maximum plasma concentration, *AUC*_*last*_ area under curve measured until the last data point, *AUC*_*INF_obs*_ area under curve from dosing time extrapolated to infinity based on the last observed concentration, *CL* clearance, *MRT*_*INF_obs*_ mean residence time from dosing time extrapolated to infinity, based on the last observed concentration, *Vss*_*_obs*_ steady-state volume of distribution based on the last observed concentration, *F* fraction absorbed (bioavailability).

^a^*n* = 3 in each dose group.

**Table 3:** In vivo toxicity of BE-33.

Administration	Species	Dosage (mg/kg)	Number of animals	Frequency	Results
Tail vein injection	C57BL/6	5/10/50	5/group	Repeat dose	No significant changes in body weight were observed with up to 50 mg/kg of BE-33 compared with the control group, however, in the 50 mg/kg administration group, a pronounced decrease in locomotor activity was observed.

## Discussion

### Physiological relevance of 2-E channels in SARS-CoV-2 infection

Comprehensive evidence supported that 2-E channels could function physiologically in SARS-CoV-2 production as well as pathogenesis. First, the mRNA level of 2-E in SARS-CoV-2-infected Vero E6 and Calu-3 cells was able to induce cell death in the heterologous expression system (Fig. 2). Second, a dominant negative mutation lowering 2-E channel activity attenuated lethality of the channel and SARS-CoV-2 production (Fig. 4). Last but not least, pharmacological inhibition of 2-E channel activity by a channel inhibitor BE-33 greatly attenuated both SARS-CoV-2 viral titer and pathogenesis of SARS-CoV-2 infection in hACE-2 mouse models (Fig. 6). Collectively, these results suggest a broader physiological relevance of 2-E channels in SARS-CoV-2 infection.

Emerging lines of evidence are pointing towards the fact that ion homeostasis is not only critical for the viability of host cell, but also an integral part of virus life cycle.^27,45^ Particularly, viral ion channels disturb the electrochemical gradient of host cell and play important roles in the assembly and release of mature virus particles from infected cells.^46^ Deletion of the gene encoding channels in the virus genome significantly reduces the formation of viral progeny and pathogenicity.^27^ In addition to monovalent potassium and sodium, 2-E channels were also permeable to divalent calcium and magnesium. Calcium has been found to promote virus entry into host cells and is essential for Ebola and SARS-CoV-2 infection.^47–49^ A recent research found that, instead of using the conventional biosynthetic secretory pathway, SARS-CoV-2 traffic to the lysosomes for egress by lysosomal exocytosis, resulting in lysosome deacidification. Consistent with our findings, the structure of 2-E channels supported that both N- and C-terminal halves are sensitive to pH change.^29,50^ The physiological relevance of the pH sensitivity of 2-E channels is worth investigating further. Alanine (A) scanning strategy was conducted to establish a relationship among the ion channel function, cell death viability and virus replication. As a result, several interesting observations were found. Substitutions of residues Phe4 or Thr11 with alanine weaken the channel activity and the capability of killing cells. Noticeably, the single-channel currents mediated by the mixture of mutant (2-E^T11A^) and WT (2-E) channels tend towards the mutant channel currents, suggesting a dominant negative role of the mutant. Consistently, we found less cell death in cells co-expressing 2-E^T11A^ and 2-E, which was not due to the decrease of protein expression level. Importantly, in a SARS-CoV-2 infection model, expression of the dominant negative mutant 2-E^T11A^ suppressed the virus replication significantly, suggesting that the 2-E channel activity is critical for virus survival and reproduction. Although how 2-E channels modulate ion homeostasis across membranes remains unclear, it is generally believed that this type of channel is needed for the virus production and maturation.^37^ An engineered SARS-CoV-2 virus with dominant negative mutations or lacking 2-E gene using reverse genetics would provide more evidence for validating the essential roles of 2-E in the virus life cycle.

### 2-E channels attack host cells and trigger inflammation as an offensive virulence factor

We found that 2-E protein may act as an “offensive” virulence factor carried by SARS-CoV-2, leading to the robust inflammatory responses and cell death, which may explain the clinical aggravation and death. From the morphology photos, like blowing balloon, 2-E-mediated cell death eventually leads to membrane rupture eventually. Upon SARS-CoV-2 membrane fusion with target cell membrane mediated by S protein and hACE-2 interaction and assisted by cofactors such as transmembrane protease serine 2 (TMPRSS2), Furin and Elastase, 2-E channel could immediately function to initiate the virulent factor and weaken the host cell defense by disturbing its ion homeostasis.^16,51^ MLKL and GSDMD, two executors of cell death, attract increasing attention recently, both disrupting plasma membrane and causing cell explosion through ion imbalance.^30,31^ In addition, it has been demonstrated that depletion of intracellular potassium as a common denominator for promoting cytokine secretion and aggravating cell death.^30,52^ These studies emphasized the importance of ion channel in cell death and inflammation.

According to clinical reports, viral replication and cytolysis are the first onset of COVID-19 infection.^53^ As the disease progresses, viral replication decrease correlates with the onset of immunoglobulin G (IgG) conversion. However, the clinical symptoms could still worsen and an over-exuberant host response leads to more severe inflammation, alveolar lung damage and other typical features during ARDS. This deterioration cannot be explained by controlled viral replication.^6,14,54^ Moreover, the severity of COVID-19 patients is linked to electrolyte imbalance, including reduced serum concentrations of potassium, sodium and calcium.^55^ However, the mechanisms of inflammation activation and electrolyte disturbance in SARS-CoV-2 infection have not yet been revealed. As seen in Fig. 1, we recorded the single-channel current mediated by the purified 2-E proteins, supporting 2-E as an ion channel permeable to potassium, sodium, calcium and magnesium. Defining 2-E as ion channels may help to unravel pathogenic mechanisms underlying how hypokalemia exacerbates ARDS.^44^ A large number of SARS-CoV-2 particles, 2-E and other DAMPs can be released simultaneously because of the sudden cell death induced by 2-E channels. Coincidently, some COVID-19 patients may worsen rapidly to sudden stroke from mild symptoms.^56,57^

### Inhibition of 2-E channel is a promising antiviral strategy and its inhibitor BE-33 is a promising anti-COVID-19 drug candidate

The lack of efficient antiviral drugs for SARS-CoV-2 has prompted the urgent need for developing new therapeutic development for COVID-19. Several options can be envisaged to control or prevent emerging infections of COVID-19, including vaccines, monoclonal antibodies, oligonucleotide-based therapies, peptides, interferon therapies and small-molecule drugs.^58^ The progress of discovering antiviral agents has been carried out actively and obtained some achievements recently, including an old drug dalbavancin and a potent neutralizing antibody BD-368-2.^59,60^ However, to date, remdesivir targeting RNA dependent RNA polymerase (RdRp) is the only approved drug for the COVID-19 treatment.^1,61^ Based on therapeutic experience with SARS-CoV and MERS-CoV, the research and development of drugs are mainly aimed at two aspects now.^62^ First is to target the virus itself, including various substrates, enzymes and viral surface proteins that inhibit the life cycle of the virus. For example, mycophenolic acids, broad-spectrum antiviral drugs inhibit the synthesis of nucleosides,^63^ lopinavir, 3C-like protease (3CLpro) inhibitor, decreases viral replication,^64^ peptides and monoclonal antibodies target viral dendritic proteins.^60,65,66^ Controlling the inflammatory response may be as important as inhibiting the viral proliferation and thus another strategy is to target host cell.^2,12,67,68^ Rational design of antiviral drug combo is a potential strategy for treating COVID-19, but it lacks solid clinical evidence. Therefore, finding highly effective drugs for the treatment is currently a top priority.

A new class of 2-E channel inhibitors exhibiting antiviral activity both in vitro and in vivo were identified in the current study. In the hACE-2 mice infection model, BE-33 suppressed SARS-CoV-2 production and limited inflammation in the lung. Importantly, in both preventive and therapeutic models, viral replication and histopathological injuries caused by SARS-CoV-2 infection were significantly inhibited by BE-33 administration. Given its relatively high safety and good PK properties, our data indicate that BE-33 is a promising anti-COVID-19 drug candidate. To our best knowledge, there are very few small molecules showing antiviral activity in terms of both virus loading and inflammation in the lungs in vivo. For example, in the ferret model, both hydroxychloroquine and lopinavir/ritonavir failed to reduce virus loading or titers in the respiratory tissues.^69^ Being the antiviral drug approved by FDA and widely used for the treatment of COVID-19, remdesivir cannot reduce the virus loading in the respiratory tract in rhesus macaques model.^70^ In contrast, the 2-E channel inhibitor BE-33 significantly reduced the virus loading in all parts of the infected lung when the drug was administrated after infection (Fig. 6c, right panel). Inspiringly, in addition to BE-33, some old drugs, such as dalbavancin, plitidepsin, mefloquine, and ganoderma lucidum (RF3), exhibited good prophylactic effects on both viral replication and lung injuries in some recent studies.^59,71,72^ The IL-6 and TNF-α serum levels were thought to be independent from the virus loadings and could act as a significant predictor of disease severity and death.^69^ Interesting to note, the IL-6 levels were significantly reduced in the mice treated with the channel inhibitor. In the absence of effective antiviral drugs against SARS-CoV-2, the favorable therapeutic efficacy observed of BE-33 in mice shed light on the potential of 2-E channel inhibitors. No mutation in 2-E has been reported so far. In addition, 2-E proteins are highly conserved among β-coronaviruses and some of them have been validated to be able to form channels in our study and other studies, suggesting the E channel may represent a broad-spectrum antiviral drug target (Supplementary information, Fig. S5).^22,28,34,73,74^ Therapies that inhibit virus life cycle and regulate dysfunctional immune responses may synergize to block pathologies. Given that 2-E can function as ion channels on the viral membranes, similar to how they function in host cells, we propose that 2-E channel may represent a new class of dual-function targets against SARS-CoV-2.

## Materials and methods

### Plasmids and mutagenesis

Wild-type SARS-CoV-2-E sequences were synthesized by the Beijing Genomics Institute (BGI, China). Vector pET28a was used for protein purification, vector pcDNA5 and pcDNA3.1 were used for cell survival assay and cell imaging. Point mutations were generated using sited-directed mutagenesis and confirmed by sequencing.

### Cell culture and treatment

HEK293, MCF-7, CaCO-2, Vero E6, HeLa, HepG2, SH-SY5Y, Calu-3 cells were grown in 90% DMEM basal medium (Gibco, USA) supplemented with 10% fetal bovine serum (FBS, Gibco, USA), 2 mM _L_-glutamine and 100 units/mL penicillin/streptomycin (Gibco, USA). 1% NEAA (Gibco, USA) was added in above medium for A498 cells culture. Besides, HCT116 and HT-29 cells were grown in McCoy’s 5 A basal medium (Gibco, USA), PC3 and A549 cells were grown in RPMI-1640 basal medium (Hyclone, USA) and CHO cells were grown in DMEM/F-12 basal medium (Gibco, USA) supplemented as above. 16HBE cells were grown in KM (ScienCell, USA) medium. For cell viability assay and western blot, cells were transfected with 3200 ng or 200 ng of SARS-CoV-2-E plasmids for 6-well or 96-well by using Lipo3000 transfection reagent according to the company’s instructions (Thermo Fisher, USA). For cytotoxicity assay, Vero E6 cells were seeded in 96-well plates with 15,000 cells per well overnight. For cell protection assay, compounds were added after transfected for 10 h. For antivirus assay, compounds were diluted with medium to appropriate concentrations, a volume of 100 μL was added to each well and then incubated for 24 h.

### Cell viability and cytotoxicity assay

Cell viability and Cytotoxicity were measured using the CCK-8 kit (40203ES60, Yeasen, China). Assays were performed according to the manufacturer’s instructions. Measure the absorbance at 450 nm with Thermo Scientific Microplate Reader (Thermo Fisher Scientific Inc., USA).

### Western blot assay and antibodies

Proteins were resolved in 12% SDS-PAGE, transferred to PVDF membranes (GE), and incubated with primary antibodies against HA-Tag (C29F4) (3724, CST, USA), GAPDH (30201ES20, Yeasen, China), mCherry (ab125096, Abcam, UK), α-Tublin (11224-1-AP, Proteintech, USA), his (30404ES60, Yeasen, China). Second antibodies are peroxidase-Conjugated Goat Anti-Rabbit IgG (H+L) (33101ES60, Yeasen, China) and Peroxidase AffiniPure Goat Anti-Mouse IgG (H+L) (33201ES60, Yeasen, China).

### Flow cytometry of cell death

After treatment, cells were detached, collected by centrifugation and resuspended in 1× binding buffer containing 100 µg/mL propidium iodide (PI) and Annexin V-FITC (1:20, V13242, Life, USA) and incubated at room temperature for 15 min in the dark. Subsequently, 400 μL of 1× binding buffer was added and cells were kept on ice. Cells were analyzed by flow cytometry using BD FACSCALIBUR 4 (BD COMPANY, USA). The percentages of differently labeled cells were calculated by FlowJo 7.6.

### Imaging

Two ways were used to examine cell death morphology. Either, cells were seeded as 2 × 10^5^ cells per well in the 6-well plates (30720113, Thermo Fisher Scientific Inc., US). After transfection for 24 h, cells were captured using Leica TCS-SP8 STED system (Leica Microsystems, DE) with a 40× phase difference objective. All image data shown are representative of at least three randomly selected fields. Or, Vero E6 cells were seeded as 1 × 10^4^ cells per well in 24-well plate overnight. The next day, the culture medium was replaced with 2% FBS DMEM. Then SARS-CoV-2 viruses were added at MOI = 0.1, with no infection as mock. At 48 h and 72 h, pictures of three visions were captured from each well by a fluorescence microscope (Olympus, Japan) at bright field.

### ELISA

Cell culture supernatants and serum were assayed for mouse IL-6 (VAL604, R&D Minnesota, USA), mouse TNF-α (VAL609, R&D Minnesota, USA), mouse IL-10 (VAL605, R&D Minnesota, USA), mouse IL-1α (VAL601, R&D Minnesota, USA), mouse IFN-γ (VAL 607, R&D Minnesota, USA), mouse IL-2 (abs520002, Absin, China) according to the manufacturer’s instructions.

### qRT-PCR Analysis

Total RNAs were extracted from cells and tissues using Trizol (Invitrogen, USA) and all total Nucleic Acid Isolation Kit (Ambion Inc., USA), following the manufacturer’s instruction. The experiment was performed at least three times using SYBR Green Master Mix (11184ES03, Yeasen, China) to quantify the mean values of delta Ct and SEM (Standard Error of Mean). The primers used for quantification were listed in Supplementary information, Table S1.

### Protein purification

The gene encoding full-length SARS-CoV-2 E protein (NCBI reference sequence YP_009724392.1, residues 1–75) was synthesized in BGI (China). For expression of full-length SARS-CoV-2-E protein, a 6× His tag was introduced at the C-terminus of 2-E gene in pET28a vector. The plasmid was transfected into *E. coli* BL21 (DE3) pLysS competent cells (CD701-02, TransGen Biotech, China) to express the fusion protein. A single colony’s DNA sequence was verified by sequencing (BGI, China). Briefly, this single colony was amplificated, starting in 50 mL LB medium with 50 μg/mL ampicillin and the cells were grown at 37 °C with shaking at 220 rpm. The starter culture was used to inoculate 10 L of LB medium with 50 μg/mL ampicillin. Cells were grown at 37 °C with shaking at 220 rpm until an optical density at 600 nm (OD600) of 0.6–0.8 was reached. Protein expression was then induced by 0.5 mM IPTG (isopropyl-b-D-1-thiogalactopyranoside, 10902ES60, Yeasen, China) at 22 °C for 16–18 h. The cell pellets were harvested by centrifugation at 4 °C and 4000 rpm for 20 min, and resuspended in binding buffer (150 mM NaCl, 20 mM Tris-base, 1 mM DTT, 1% PMSF, 1× cocktail, pH 8.0). The resuspended cells were lysed by high-pressure homogenizer under 800–900 bar for 3–8 min at 4 °C. The soluble fraction of the cell lysate was separated from the cell fragments by centrifugation at 10,000× *g* for 30 min and the supernatant was filtered through a 0.22 μM filter to remove particles. The Ni-NTA resin (20502ES60, Yeasen, China) was pre-equilibrated with TBS buffer (150 mM NaCl, 20 mM Tris-base, pH 8.0). The clarified supernatant was loaded on 5 mL Ni-NTA resin for 4 h to separate the 2-E-6× His fusion protein from the *E. coli* proteins. The column was washed with 10-fold column volume of the imidazole concentration gradient at 20 mM, 30 mM, 50 mM in TBS, respectively. After washing, the 2-E fusion protein was eluted with 300 mM imidazole in TBS. The 2-E fusion protein was then purified by gel filtration chromatography using a Superdex 75 Increase 10/300 gel filtration chromatography (29148721, GE Healthcare, USA) pre-equilibrated with TBS. Fractions containing the 2-E protein were collected. Last, we used the endotoxin removal agarose resin (20518ES10, Yeasen, China) to remove the endotoxin.^75,76^ After balancing the agarose resin by TBS, the purified protein was loaded on the resin at the speed of 0.25 mL/min. The elution was collected for concentration and application in electrophysiology recording, macrophage cell immunity experiments and animal model.

### LC-MS/MS analysis

All liquid chromatography-tandem mass spectrometry (LC-MS/MS) experiments were carried on an online setup consisting of an Easy-nano LC system and a Q-Exactive HF mass spectrometer (Thermo, Bremen, Germany). The peptides were loaded on in-house packed column (75 μm × 200 mm fused silica column with 3 μm ReproSil-Pur C18 beads, Dr. Maisch GmbH, Ammerbuch, Germany) and separated with a 60-min gradient at a flow rate of 300 nL/min. Solvent A contained 100% H_2_O and 0.1% formic acid, Solvent B contained 100% acetonitrile and 0.1% formic acid. The spray voltage was set at 2500 V in positive ion mode and the ion transfer tube temperature was set at 275 °C. The MS1 full scan was set at a resolution of 60,000 at m/z 200, AGC target 3e6 and maximum IT 20 ms, followed by MS2 scans at a resolution of 15,000 at m/z 200, AGC target 1e5 and maximum IT 100 ms. The precursor ions were fragmented by higher energy collisional dissociation (HCD). Isolation window was set at 1.6 m/z. The normalized collision energy (NCE) was set at NCE 27%, and the dynamic exclusion time was 30 s. Precursors with charge 1, 7, 8 and > 8 were excluded for MS2 analysis.

The MS raw data were analyzed with MaxQuant (http://maxquant.org/, 1.6.7.0). Oxidized methionine and protein N-term acetylation were set as variable modifications. Trypsin/P or chymotrypsin was selected as the digestive enzyme with two potential missed cleavages. The false discovery rate (FDR) for peptides and proteins was rigorously controlled < 1%.

### Planar lipid bilayers recording

The purified proteins were incorporated into lipid bilayers to test their functionality.^77^ All the lipids were bought from Avanti (Avanti Polar Lipids, USA). Proteins were added to *cis* side and the buffer contained 500 mM KCl in *cis*/50 mM KCl in *Trans*, and all solutions were buffered by 5 mM HEPES, pH 6.35. Membrane currents were recorded under voltage-clamp mode using a Warnner bilayer clamp amplifier BC-535 (Warner Instruments, USA), filtered at 1–2 kHz. The recording frequency was 10 kHz. The currents were digitized using pClamp 10.2 software (Molecular Devices, US). Data are presented as means ± SEM. The single-channel conductance and open time were determined by fitting to Gaussian functions (bin width = 0.25 pA) or to single or bi-exponential equations. Opening time less than 0.5–1.5 ms was ignored. The equilibrium potential was calculated using the Nernst equation and Goldman–Hodgkin–Katz flux equation.

### SPR assay

Biacore T200 instruments (GE Healthcare) were used to evaluate the binding affinity of compounds to 2-E protein via SPR, as previously described.^78^ Briefly, 2-E protein was immobilized on the surface of CM5 chip by using amine-coupling approach at a flow rate of 10 μL/min in 10 mM sodium acetate buffer (pH 4.5). The sensor surface was activated with a 7 min injection of the mixture of 50 mM N-hydroxysuccinimide (NHS) and 200 mM 1-ethyl-3-(3-dimethylaminopropyl) carbodiimide (EDC). Then 50 μg/mL of Tau protein was injected to reach the target level of around 1400 RU and the surface was blocked with 1 M ethanolamine, pH 8.5. Serial concentrations (typically 1.5625 μM, 3.125 μM, 6.25 μM, 12.5 μM, 25 μM, 50 μM, 100 μM, 200 μM) of compounds were injected into the flow system and analyzed for 90 s, and the dissociation was 120 s. All binding analysis was performed in phosphate buffered saline (PBS) with 0.05% (v/v) Tween-20 and 1% DMSO, pH 7.4, at 25 °C. Prior to analysis, double reference subtractions were made to eliminate bulk refractive index changes, injection noise, and data drift. The binding affinity was determined by fitting to a Langmuir 1:1 binding model within the Biacore Evaluation software (GE Healthcare).

### Viral inhibition assay

SARS-CoV-2 (nCoV-2019BetaCoV/Wuhan/WIV04/2019) was preserved at Wuhan Institute of Virology, Chinese Academy of Sciences (CAS). It was propagated and titrated with Vero E6 cells, and its associated operations were performed in a biosafety level 3 (BSL-3) facility. For viral inhibition assay, Vero E6 cells seeded in 48-well plate with 50,000 cells per well overnight were firstly pre-treated with gradient-diluted compounds for 1 h, followed by adding SARS-CoV-2 (MOI = 0.01) and incubating for 1 h at 37 °C. After that, the virus–drug mixture was completely removed, and the cells were washed twice with PBS followed by adding fresh medium with compounds. 24 h later, viral RNA was extracted from cell supernatants with Mini BEST Viral RNA/DNA Extraction Kit (Takara, Japan) according to the instructions, then reverse transcribed with Prime ScriptTM RT reagent Kit with gDNA Eraser (Takara, Japan). Viral genome copies were quantified with Takara TB Green^®^ Premix Ex Taq™ II (Takara, Japan) by a standard curve method on ABI 7500 using a pair of primers targeting S gene. The forward primer: 5′-CAATGGTTTAACAGGCACAGG -3′, the reverse primer: 5′-CTCAAGTGTCTGTGGATCACG-3′.

### Animal model

SARS-CoV-2-E protein induced mouse model was established by conducting tail vein injection of purified protein (25 mg/kg body weight) in 8-week-old mice. Male C57BL/6 mice were obtained from Shanghai SLAC Laboratory Animal Co., Ltd. All animal procedures were performed in accordance with the National Institutes of Health Guide for the Care and Use of Laboratory Animals, under protocols approved and strictly followed by the Institutional Animal Care and Use Committees (IACUC).

The hACE-2 mice were kindly provided by Dr. Jiekai Chen and were maintained under specific-pathogen-free (SPF) conditions in ABSL-3 and were used at 14 to 18 weeks. All animals were allowed free access to water and diet and provided with a 12 h light/dark cycle. All of the animal experiments were performed following recommendations in the Guide for the Care and Use of Laboratory Animals of Kunming Institute of Zoology (KIZ), CAS. The Institutional Committee for Animal Care and Biosafety at KIZ, CAS, approved works in ABSL-3 (Approval ID: SMKX--tz-20200415-03).

### Virus titration for animal model

The SARS-CoV-2 strain (accession number: NMDCN0000HUI) was kindly provided by Guangdong Provincial Center for Disease Control and Prevention, Guangdong Province of China. The virus was propagated and titrated in Vero E6 cells, which were cultured in DMEM supplemented with 10% FBS. The sequence of virus can be accessed in the China National Microbiology Data Center (Accession No. NMDCN0000HUI).

### Mouse infection and sample collection

The hACE-2 mice (*n* = 36) were anesthetized with isoflurane (RWD Life Science), and then intranasally infected with 2 × 10^6^ TCID_50_ of SARS-CoV-2 in 30 μL of DMEM, respectively. For the mock control, the mice intranasally received 30 μL of DMEM. All mice were observed and their body weights were monitored daily until sacrifice. Tissue samples were collected from sacrificed mice at the indicated time points post-infection. Briefly, mice were deeply anesthetized with isoflurane and sacrificed. Different tissues were collected and stored in –80 °C freezer or 4% PFA until use.

### Viral loading measurement

Briefly, tissue samples were dissolved in 1 mL of TRIzol™ Reagent (Thermo, USA) and then subjected to RNA extraction according to the manufacture’s protocol. The extracted RNA was used to measure the nucleoprotein N gene copies of SARS-CoV-2 using THUNDERBIRD^®^ Probe One-step qRT-PCR Kit (Toyobo) following the manufacture’s protocol. Results were expressed as number of copies/μg tissue total RNA. The primer sequences for qRT-PCR were as follows: forward primer: 5′-GGGGAACTTCTCCTGCTAGAAT-3′; reverse primer: 5′-CAGACATTTTGCTCTCAAGCTG-3′.

### Histology/immunofluorescence

Tissues from virally infected mice were fixed in 4% PFA for at least 7 days, and then were paraffin embedded and cut into 3-μm sections following the standard procedure. The sections were stained with H&E, and examined by light microscopy. The pathological score was assessed based on the degree of lung tissue lesions including alveolar septal thickening, hemorrhage, inflammatory cells infiltration, and consolidation. The semiquantitative assessment was performed as follows: we scored 0 when no alveolar septal thickening was observed, scored 1 when alveolar septal thickening was very mild and the area of alveolar septal thickening, hemorrhage and inflammatory cells infiltration was less than 10%, scored 2 when alveolar septal thickening was mild and the area of alveolar septal thickening, hemorrhage and inflammatory cells infiltration was 10%–25%, scored 3 when alveolar septal thickening was moderate and the area of alveolar septal thickening, hemorrhage and inflammatory cells infiltration was 25%–50%, scored 4 when alveolar septal thickening was marked and the area of alveolar septal thickening, hemorrhage, inflammatory cells infiltration and consolidation was 50%–75%, and scored 5 when alveolar septal thickening was very marked and the area of alveolar septal thickening, hemorrhage, inflammatory cells infiltration, and consolidation was greater than 75%. The images were taken by Leica DM 6B microscope.

### In vivo pharmacokinetics in mice

Pharmacokinetic studies were performed in male C57BL/6 mice weighing 16–20 g (*n* = 3). Pharmacokinetic parameters were obtained by single intravenous injection (2.5 mg/kg) or oral administration of BE-33 (10 mg/kg), which was dissolved in a mixed solution (po: DMSO/0.5% HPMC (v/v = 5/95). iv: DMSO/EtOH/PEG300/0.9%NaCl (v/v/v/v = 5/5/40/50)). Heparinized samples of blood were collected at 0.083 h, 0.25 h, 0.5 h, 1 h, 2 h, 4 h, 8 h, and 24 h after intravenous dosing and 0.25 h, 0.5 h, 1 h, 2 h, 4 h, 6 h, 8 h, and 24 h after oral dosing, respectively. Plasma was separated by centrifugation and stored frozen at –20 °C until subsequent analysis. Bioanalysis of samples was analyzed by LC-MS/MS. For tissue distribution assay, 12 healthy ICR male mice, weighing 20–25 g, fasted for 12 h before the test, free to drink water. At 0.25 h, 1 h, 4 h and 16 h after oral administration, the abdominal aorta was sacrificed by bloodletting, 3 male mice at each time point. 0.5 mL of whole blood was collected from each animal, placed in heparinized tubes, centrifuged at 15,000 rpm for 5 min, and plasma was separated and stored frozen in a refrigerator at –20 °C. After the animals were sacrificed, the brain, liver, lung, kidney, and Duodenum were dissected, and the residual blood was washed with ice physiological saline. Another 2 male mice were used as control. The concentration of each compound in plasma and tissue was determined by LC-MS/MS.

### Toxicity study for 1 week of BE-33

Toxicity study was performed in SPF C57/BL6 male mice weighing 20–25 g. Dose range toxicity studies for one week were performed. C57/BL6 mice were assigned to six groups which contained one vehicle group and five intravenous injection (0.2 mL/min) groups (3–6 mice per group), the dosage of BE-33 was 5, 10, 50 mg/kg, respectively. All animals were clinically observed at least once a day for at least 7 days for toxic signs including bodyweight, food and water intake, rectal temperature and hematology. At the end of the experiment, samples of heart, liver, spleen, lung, kidney and administration site were collected.

## Supplementary information

Supplementary information, Fig. S1

Supplementary information, Fig. S2

Supplementary information, Fig. S3

Supplementary information, Fig. S4

Supplementary information, Fig. S5

Supplementary information, Fig. S6

Supplementary information, Fig. S7

Supplementary information, Fig. S8

Supplementary information, Fig. S9

Supplementary information, Fig. S10

Supplementary information, Fig. S11

Supplementary information, Fig. S12

Supplementary information, Fig. S13

Supplementary information, Fig. S14

Supplementary information, Fig. S15

Supplementary information, Fig. S16

Supplementary Methods

Supplementary information, Table S1

## References

[1] Guan WJ (2020). Clinical characteristics of coronavirus disease 2019 in China. N. Engl. J. Med..

[2] Mehta P (2020). COVID-19: consider cytokine storm syndromes and immunosuppression. Lancet.

[3] Zhang H (2020). Histopathologic changes and SARS-CoV-2 immunostaining in the lung of a patient with COVID-19. Ann. Intern. Med..

[4] Xu Z (2020). Pathological findings of COVID-19 associated with acute respiratory distress syndrome. Lancet Respir. Med..

[5] Azkur AK (2020). Immune response to SARS-CoV-2 and mechanisms of immunopathological changes in COVID-19. Allergy.

[6] Cao X (2020). COVID-19: immunopathology and its implications for therapy. Nat. Rev. Immunol..

[7] Grifoni A (2020). A sequence homology and bioinformatic approach can predict candidate targets for immune responses to SARS-CoV-2. Cell Host Microbe.

[8] Peeples L (2020). News feature: avoiding pitfalls in the pursuit of a COVID-19 vaccine. Proc. Natl. Acad. Sci. USA.

[9] de Wit E, van Doremalen N, Falzarano D, Munster VJ (2016). SARS and MERS: recent insights into emerging coronaviruses. Nat. Rev. Microbiol..

[10] Zaki AM, van Boheemen S, Bestebroer TM, Osterhaus AD, Fouchier RA (2012). Isolation of a novel coronavirus from a man with pneumonia in Saudi Arabia. N. Engl. J. Med..

[11] Zhong NS (2003). Epidemiology and cause of severe acute respiratory syndrome (SARS) in Guangdong, People’s Republic of China, in February, 2003. Lancet.

[12] Merad M, Martin JC (2020). Pathological inflammation in patients with COVID-19: a key role for monocytes and macrophages. Nat. Rev. Immunol..

[13] Tay MZ, Poh CM, Rénia L (2020). The trinity of COVID-19: immunity, inflammation and intervention. Immunity.

[14] Blanco-Melo D (2020). Imbalanced host response to SARS-CoV-2 drives development of COVID-19. Cell.

[15] Kim D (2020). The architecture of SARS-CoV-2 transcriptome. Cell.

[16] Hoffmann M (2020). SARS-CoV-2 cell entry depends on ACE2 and TMPRSS2 and is blocked by a clinically proven protease inhibitor. Cell.

[17] Lan J (2020). Structure of the SARS-CoV-2 spike receptor-binding domain bound to the ACE2 receptor. Nature.

[18] Wrapp D, Wang N (2020). Cryo-EM structure of the 2019-nCoV spike in the prefusion conformation. Science.

[19] Yoshimoto FK (2020). The proteins of severe acute respiratory syndrome coronavirus-2 (SARS CoV-2 or n-COV19), the cause of COVID-19. Protein J..

[20] Cong, Y. et al. Nucleocapsid protein recruitment to replication-transcription complexes plays a crucial role in coronaviral life cycle. *J. Virol.* **94**, e01925-19 (2020). 10.1128/JVI.01925-19 PMC6997762 31776274

[21] Toto A (2020). Comparing the binding properties of peptides mimicking the Envelope protein of SARS-CoV and SARS-CoV-2 to the PDZ domain of the tight junction-associated PALS1 protein. Protein Sci..

[22] Schoeman D, Fielding BC (2019). Coronavirus envelope protein: current knowledge. Virol. J..

[23] Liao Y, Yuan Q, Torres J, Tam JP, Liu DX (2006). Biochemical and functional characterization of the membrane association and membrane permeabilizing activity of the severe acute respiratory syndrome coronavirus envelope protein. Virology.

[24] Pervushin K (2009). Structure and inhibition of the SARS coronavirus envelope protein ion channel. PLoS Pathog..

[25] Verdiá-Báguena C (2013). Analysis of SARS-CoV E protein ion channel activity by tuning the protein and lipid charge. Biochim. Biophys. Acta.

[26] Westerbeck, J. W. & Machamer, C. E. The infectious bronchitis coronavirus envelope protein alters Golgi pH to protect the spike protein and promote the release of infectious virus. *J. Virol.* **93**, e00015-19 (2019). 10.1128/JVI.00015-19 PMC6532078 30867314

[27] Nieto-Torres JL (2014). Severe acute respiratory syndrome coronavirus envelope protein ion channel activity promotes virus fitness and pathogenesis. PLoS Pathog..

[28] Li S (2019). Regulation of the ER stress response by the ion channel activity of the infectious bronchitis coronavirus envelope protein modulates virion release, apoptosis, viral fitness, and pathogenesis. Front. Microbiol..

[29] Mandala, V. S. et al. Structure and drug binding of the SARS-CoV-2 envelope protein transmembrane domain in lipid bilayers. *Nat. Struct. Mol. Biol.* **27**, 1202–1208 (2020). 10.1038/s41594-020-00536-8 PMC7718435 33177698

[30] Ding J (2016). Pore-forming activity and structural autoinhibition of the gasdermin family. Nature.

[31] Xia B (2016). MLKL forms cation channels. Cell Res..

[32] Gong YN (2017). ESCRT-III acts downstream of MLKL to regulate necroptotic cell death and its consequences. Cell.

[33] Wang Y (2017). Chemotherapy drugs induce pyroptosis through caspase-3 cleavage of a gasdermin. Nature.

[34] Su S (2016). Epidemiology, genetic recombination, and pathogenesis of coronaviruses. Trends Microbiol..

[35] Wang C, Horby PW, Hayden FG, Gao GF (2020). A novel coronavirus outbreak of global health concern. Lancet.

[36] Pizzorno A (2020). Characterization and treatment of SARS-CoV-2 in nasal and bronchial human airway epithelia. Cell Rep. Med..

[37] Nieto-Torres JL (2015). Severe acute respiratory syndrome coronavirus E protein transports calcium ions and activates the NLRP3 inflammasome. Virology.

[38] Bao, L. et al. The pathogenicity of SARS-CoV-2 in hACE2 transgenic mice. *Nature* **583**, 830–833 (2020). 10.1038/s41586-020-2312-y 32380511

[39] Noris, M., Benigni, A. & Remuzzi, G. The case of Complement activation in COVID-19 multiorgan impact. *Kidney Int.* **98**, 314–322 (2020). 10.1016/j.kint.2020.05.013 PMC7246017 32461141

[40] Hassan, A. O. et al. A SARS-CoV-2 infection model in mice demonstrates protection by neutralizing antibodies. *Cell* **182**, 744–753 (2020). 10.1016/j.cell.2020.06.011 PMC7284254 32553273

[41] Jiang RD (2020). Pathogenesis of SARS-CoV-2 in transgenic mice expressing human angiotensin-converting enzyme 2. Cell.

[42] Zhang Y-N (2020). A mouse model for SARS-CoV-2 infection by exogenous delivery of hACE2 using alphavirus replicon particles. Cell Res..

[43] Liu, F.-L. et al. Rapid generation of ACE2 humanized inbred mouse model for COVID-19 with tetraploid complementation. *Natl. Sci. Rev.* **8**, nwaa285 10.1093/nsr/nwaa285 (2021). 10.1093/nsr/nwaa285 PMC7717373 34676093

[44] Xu, L. et al. COVID-19-like symptoms observed in Chinese tree shrews infected with SARS-CoV-2. *Zool. Res.* **41**, 517–526 (2020). 10.24272/j.issn.2095-8137.2020.053 PMC7475013 32701249

[45] Nieva JL, Madan V, Carrasco L (2012). Viroporins: structure and biological functions. Nat. Rev. Microbiol..

[46] Triantafilou K, Triantafilou M (2014). Ion flux in the lung: virus-induced inflammasome activation. Trends Microbiol..

[47] Chen, X., Cao, R., & Zhong, W. Host calcium channels and pumps in viral infections. *Cells* **9**, 10.3390/cells9010094 (2019). 10.3390/cells9010094 PMC7016755 31905994

[48] Dakal TC (2021). SARS-CoV-2 attachment to host cells is possibly mediated via RGD-integrin interaction in a calcium-dependent manner and suggests pulmonary EDTA chelation therapy as a novel treatment for COVID 19. Immunobiology.

[49] Sakurai Y (2015). Ebola virus. Two-pore channels control Ebola virus host cell entry and are drug targets for disease treatment. Science.

[50] Ghosh S (2020). Beta-coronaviruses use lysosomes for EGRESS instead of the biosynthetic secretory pathway. Cell.

[51] Chen N (2020). Epidemiological and clinical characteristics of 99 cases of 2019 novel coronavirus pneumonia in Wuhan, China: a descriptive study. Lancet.

[52] Conos SA (2017). Active MLKL triggers the NLRP3 inflammasome in a cell-intrinsic manner. Proc. Natl. Acad. Sci. USA.

[53] Li H (2020). SARS-CoV-2 and viral sepsis: observations and hypotheses. Lancet.

[54] Louhaichi S (2020). Features of patients with 2019 novel coronavirus admitted in a pneumology department: the first retrospective Tunisian case series. La Tunisie Med..

[55] Lippi G, South AM (2020). Electrolyte imbalances in patients with severe coronavirus disease 2019 (COVID-19). Ann. Clin. Biochem..

[56] Ellul MA (2020). Neurological associations of COVID-19. Lancet Neurol..

[57] Qureshi AI (2020). Management of acute ischemic stroke in patients with COVID-19 infection: report of an international panel. Int J. Stroke.

[58] Sanders, J. M., Monogue, M. L., Jodlowski, T. Z. & Cutrell, J. B. Pharmacologic treatments for coronavirus disease 2019 (COVID-19): a review. *JAMA* **323**, 1824–1836 (2020). 10.1001/jama.2020.6019 32282022

[59] Wang, G. et al. Dalbavancin binds ACE2 to block its interaction with SARS-CoV-2 spike protein and is effective in inhibiting SARS-CoV-2 infection in animal models. *Cell Res.* **31**, 17–24 (2021). 10.1038/s41422-020-00450-0 PMC7705431 33262453

[60] Du S (2020). Structurally resolved SARS-CoV-2 antibody shows high efficacy in severely infected hamsters and provides a potent cocktail pairing strategy. Cell.

[61] Ferner RE, Aronson JK (2020). Remdesivir in covid-19. BMJ.

[62] Zumla, A., Chan, J. F., Azhar, E. I., Hui, D. S. & Yuen, K. Y. Coronaviruses — drug discovery and therapeutic options. *Nat. Rev. Drug Discov.* **15**, 327–347 (2016). 10.1038/nrd.2015.37 PMC7097181 26868298

[63] Chan JF (2015). Middle East respiratory syndrome coronavirus: another zoonotic betacoronavirus causing SARS-like disease. Clin. Microbiol. Rev..

[64] Dalerba P, Levin B, Thompson JL (2020). A trial of Lopinavir-Ritonavir in Covid-19. N. Engl. J. Med..

[65] Amanat F, Krammer F (2020). SARS-CoV-2 vaccines: status report. Immunity.

[66] Wang N (2021). Structure-based development of human antibody cocktails against SARS-CoV-2. Cell Res..

[67] Zhu N (2020). A novel coronavirus from patients with pneumonia in China, 2019. N. Engl. J. Med..

[68] Lu S (2021). Effective treatment of SARS-CoV-2-infected rhesus macaques by attenuating inflammation. Cell Res..

[69] Del Valle DM (2020). An inflammatory cytokine signature predicts COVID-19 severity and survival. Nat. Med..

[70] Williamson, B. N. et al. Clinical benefit of remdesivir in rhesus macaques infected with SARS-CoV-2. *Nature* **585**, 273–276 (2020). 10.1038/s41586-020-2423-5 PMC7486271 32516797

[71] White, K. M. et al. Plitidepsin has potent preclinical efficacy against SARS-CoV-2 by targeting the host protein eEF1A. *Science* **371**, 926–931 (2021). 10.1126/science.abf4058 PMC7963220 33495306

[72] Jan, J.-T. et al. Identification of existing pharmaceuticals and herbal medicines as inhibitors of SARS-CoV-2 infection. *Proc. Natl. Acad. Sci. USA* **118**, e2021579118 (2021). 10.1073/pnas.2021579118 PMC7865145 33452205

[73] Wilson L, McKinlay C, Gage P, Ewart G (2004). SARS coronavirus E protein forms cation-selective ion channels. Virology.

[74] Stodola JK, Dubois G, Le Coupanec A, Desforges M, Talbot PJ (2018). The OC43 human coronavirus envelope protein is critical for infectious virus production and propagation in neuronal cells and is a determinant of neurovirulence and CNS pathology. Virology.

[75] Yin L (2019). Functional characterization of three fish-specific interleukin-23 isoforms as regulators of Th17 signature cytokine expression in grass carp head kidney leukocytes. Fish Shellfish Immunol..

[76] Zhang A (2020). Characterization and bioactivity of grass carp (Ctenopharyngodon idella) interleukin-21: Inducible production and involvement in inflammatory regulation. Fish Shellfish Immunol..

[77] Gaburjakova, J. & Gaburjakova, M. Reconstitution of ion channels in planar lipid bilayers: new approaches. *Advances in Biomembranes and Lipid Self-Assembly* **27**, 147–185 (2018).

[78] Li J (2017). The omega-carboxyl group of 7-ketocholesteryl-9-carboxynonanoate mediates the binding of oxLDL to CD36 receptor and enhances caveolin-1 expression in macrophages. Int. J. Biochem. Cell Biol..

