# Supplementary material for: SARS-CoV-2 envelope protein causes acute respiratory distress syndrome (ARDS)-like pathological damages and constitutes an antiviral target
Source: Cell Res. 2021 Jun 10;31(8):847–60. doi: 10.1038/s41422-021-00519-4 (PMC8190750; doi:10.1038/s41422-021-00519-4)
Supplement: Supplementary file 1 — Supplementary information, Fig. S1 [file 41422_2021_519_MOESM1_ESM.pdf]

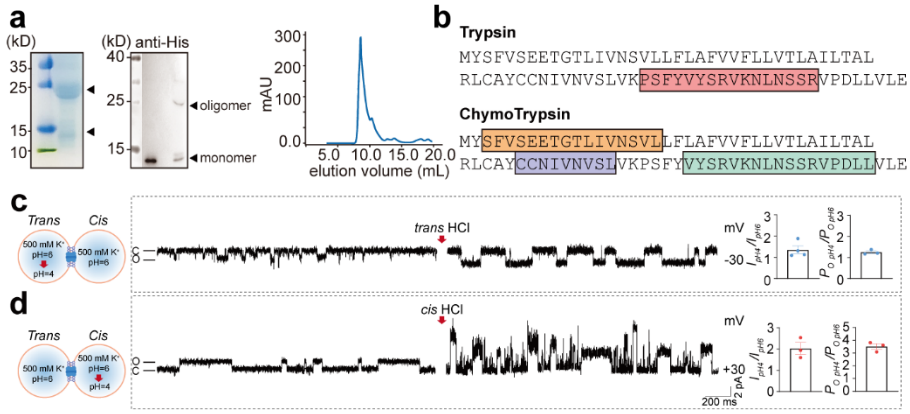

Supplementary information, Fig.S1: Purification of 2-E proteins.

a Purification of full-length 2-E protein with Ni-NTA affinity chromatography. Left: 15% SDS-PAGE gel with coomassie blue staining; Middle: Western blot probed with anti-his-tag antibody; Right: Size-exclusion chromatogram of the affinity-purified 2-E protein. Data from a Superdex 75 Increase 10/300 column are shown in blue. The elution peak probably represents 2-E protein oligomers. **b** Four peptides (red, green, orange and purple) of 2-E were detected by LC-MS/MS. Two enzymatic methods were used. **c** Titration results of different pH on a same channel. The pH of the symmetric KCl solutions (*trans : cis* = 500 :500 mM) in the *cis* side was maintained at 6 and the pH of the solution in *trans* side was titrated to 4 (red arrow). Amplitude (*I*) and open probability (*P*o) were normalized to the amplitude and open probability measured at pH 6 of both sides. **d** The pH in the *trans* side was maintained at 6 whereas the pH in *cis* side was titrated to 4 (red arrow). Amplitude (*I*) and open probability (*P*o) were normalized to the amplitude and open probability measured at pH 6 of both sides. All error bars are SEM.
