# Supplementary material for: SARS-CoV-2 envelope protein causes acute respiratory distress syndrome (ARDS)-like pathological damages and constitutes an antiviral target
Source: Cell Res. 2021 Jun 10;31(8):847–60. doi: 10.1038/s41422-021-00519-4 (PMC8190750; doi:10.1038/s41422-021-00519-4)
Supplement: Supplementary file 2 — Supplementary information, Fig. S2 [file 41422_2021_519_MOESM2_ESM.pdf]

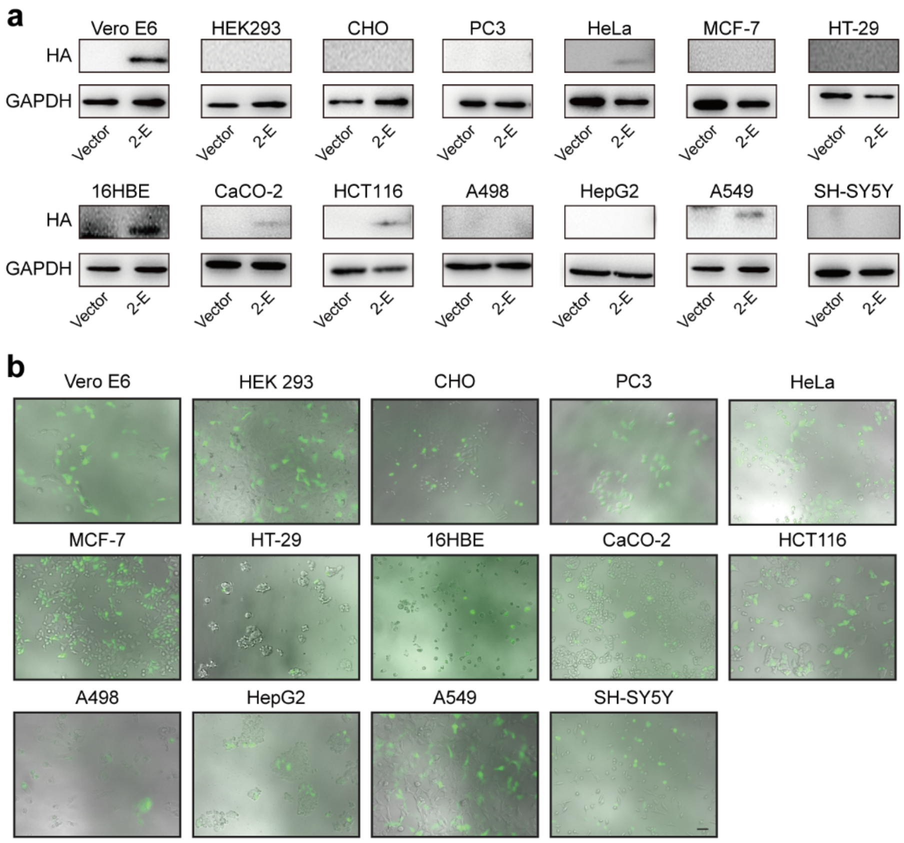

Supplementary information, Fig. S2: Expression level of 14 cell lines after 2-E transfection.

a Expression of 2-E in various cell lines. **b** Images for 14 cell lines after transfecting with GFP plasmid (Scale, 50 μm).
