# Supplementary material for: SARS-CoV-2 envelope protein causes acute respiratory distress syndrome (ARDS)-like pathological damages and constitutes an antiviral target
Source: Cell Res. 2021 Jun 10;31(8):847–60. doi: 10.1038/s41422-021-00519-4 (PMC8190750; doi:10.1038/s41422-021-00519-4)
Supplement: Supplementary file 4 — Supplementary information, Fig. S4 [file 41422_2021_519_MOESM4_ESM.pdf]

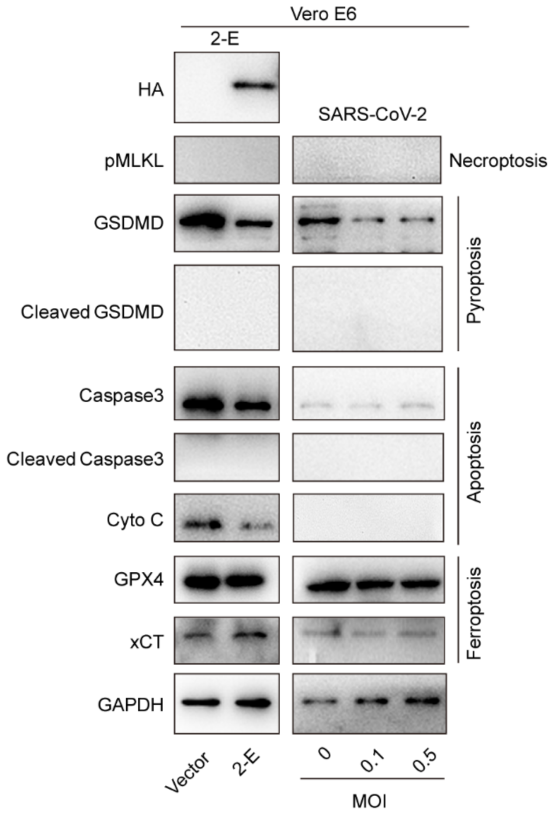

Supplementary information, Fig. S4： Immunoblotting of cell death pathway biomarkers in 2-E transfected and SARS-CoV-2 infected Vero E6 cells.
