# Supplementary material for: SARS-CoV-2 envelope protein causes acute respiratory distress syndrome (ARDS)-like pathological damages and constitutes an antiviral target
Source: Cell Res. 2021 Jun 10;31(8):847–60. doi: 10.1038/s41422-021-00519-4 (PMC8190750; doi:10.1038/s41422-021-00519-4)
Supplement: Supplementary file 5 — Supplementary information, Fig. S5 [file 41422_2021_519_MOESM5_ESM.pdf]

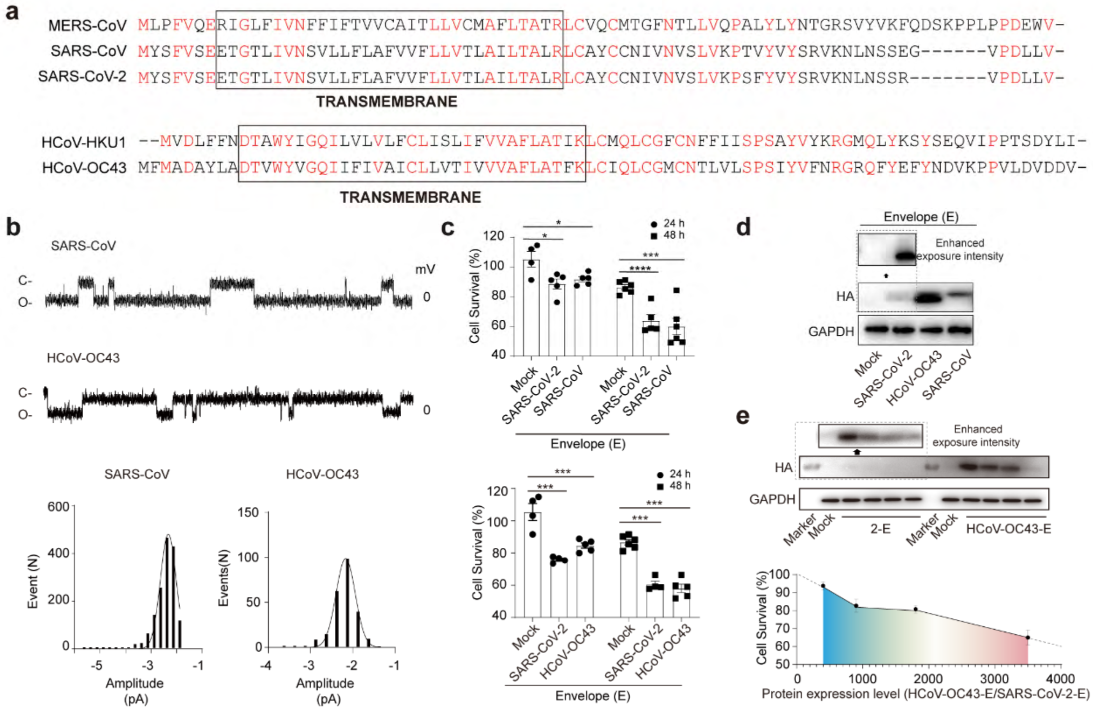

Supplementary information, Fig. S5: Comparison of different coronaviruses envelope proteins (E).

a Sequence alignment of five human β-coronaviruses E proteins. **b** Single-channel current recording of SARS-CoV-E and HCoV-OC43-E proteins after reconstitution in lipid bilayers with 50:500 mM KCl solutions (*trans* : *cis*) , PC/PS = 3:2 lipids at the indicated potentials. Protein (5-50 ng/mL) was added to the *cis* side. "C" means channel close; "O" means channel open**. c** Survival viability of Vero E6 cells under 2-E, SARS-CoV-E and HCoV-OC43-E transfection at 24 and 48 hours. **d** The expression level of 2-E-HA, SARS-CoV-E-HA and HCoV-OC43-E-HA protein in Vero E6 cells. **e** The correlation between capability of killing cell and the relative expression level of HCoV-OC43-E protein normalized by 2-E expression. $*P < 0.05$; $**P < 0.01$; $***P < 0.001$; unpaired Student's t test. All error bars are SEM.
