# Supplementary material for: SARS-CoV-2 envelope protein causes acute respiratory distress syndrome (ARDS)-like pathological damages and constitutes an antiviral target
Source: Cell Res. 2021 Jun 10;31(8):847–60. doi: 10.1038/s41422-021-00519-4 (PMC8190750; doi:10.1038/s41422-021-00519-4)
Supplement: Supplementary file 6 — Supplementary information, Fig. S6 [file 41422_2021_519_MOESM6_ESM.pdf]

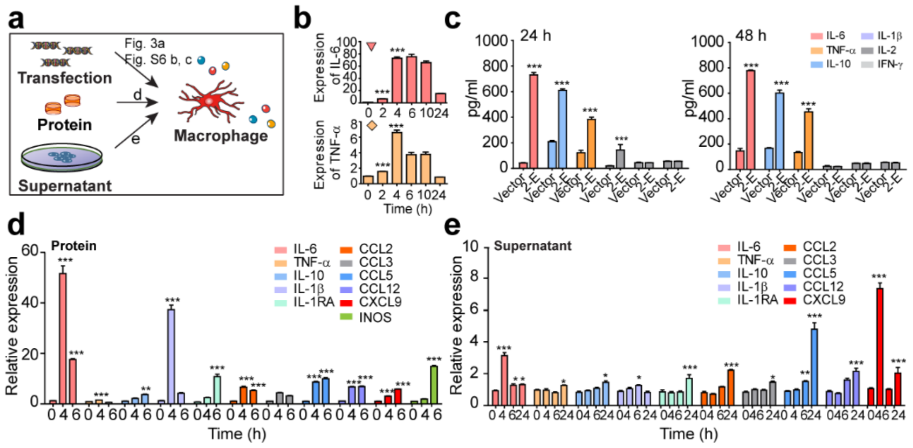

Supplementary information, Fig. S6: SARS-CoV-2-E provokes robust immune responses in vitro.

a Schematic for detecting the immune response of macrophages. **b-c** Expression of cytokines and chemokines following transfection of 2-E plasmids. **b** Expression levels of IL-6 and TNF-α at indicated time after transfection with 2-E plasmids via qRT-PCR. **c** Cytokine levels after 2-E transfected via ELISA. **d** qRT-PCR analysis of cytokine expression upon treatment with purified 2-E proteins. **e** Incubation with Vero E6 culture supernatant, measuring mRNA expression via qRT-PCR. *$P < 0.05$; **$P < 0.01$; *** < 0.001; unpaired Student’s t test. All error bars are SEM.
