# Supplementary material for: SARS-CoV-2 envelope protein causes acute respiratory distress syndrome (ARDS)-like pathological damages and constitutes an antiviral target
Source: Cell Res. 2021 Jun 10;31(8):847–60. doi: 10.1038/s41422-021-00519-4 (PMC8190750; doi:10.1038/s41422-021-00519-4)
Supplement: Supplementary file 7 — Supplementary information, Fig. S7 [file 41422_2021_519_MOESM7_ESM.pdf]

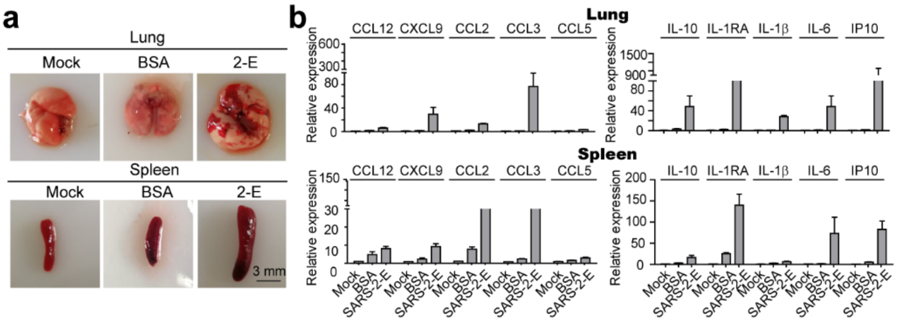

Supplementary information, Fig. S7: Bovine serum albumin did not cause acute respiratory distress syndrome (ARDS)-like damage in the lung and spleen in mice. **a** Gross pathology of lung and spleen from control mice (Mock, Tris-buffered saline (TBS) treated; Bovine serum albumin (BSA) (25 mg/kg body weight)), and model mice (2-E proteins). **b** qRT-PCR analysis of the lung and spleen tissues after injection of TBS, BSA and 2-E proteins.
