# Supplementary material for: SARS-CoV-2 envelope protein causes acute respiratory distress syndrome (ARDS)-like pathological damages and constitutes an antiviral target
Source: Cell Res. 2021 Jun 10;31(8):847–60. doi: 10.1038/s41422-021-00519-4 (PMC8190750; doi:10.1038/s41422-021-00519-4)
Supplement: Supplementary file 8 — Supplementary information, Fig. S8 [file 41422_2021_519_MOESM8_ESM.pdf]

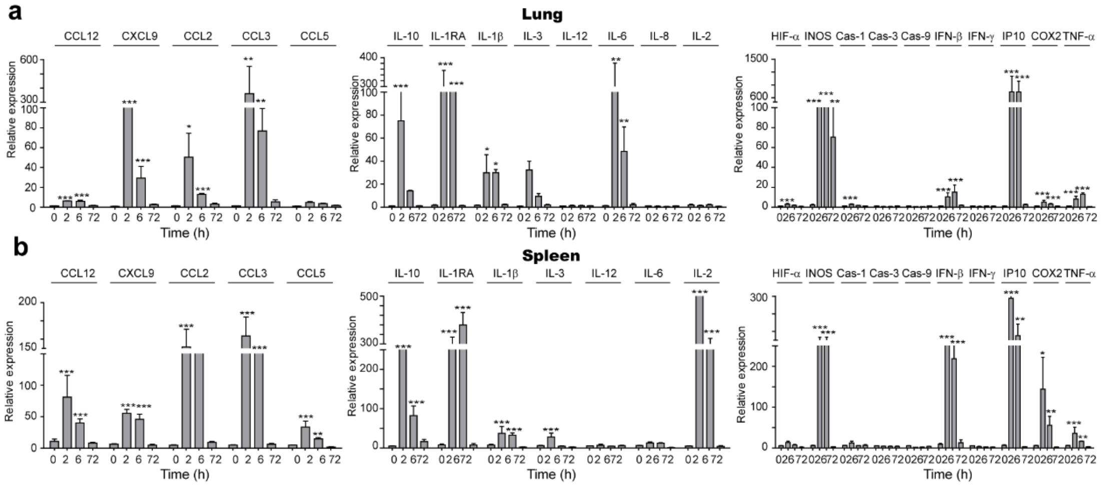

Supplementary information, Fig. S8: qRT-PCR analysis of lung and spleen tissues after injection of 2-E proteins. **P < 0.05; **P < 0.01; ***P < 0.001;* unpaired Student's t test. All error bars are SEM.
