# Supplementary material for: SARS-CoV-2 envelope protein causes acute respiratory distress syndrome (ARDS)-like pathological damages and constitutes an antiviral target
Source: Cell Res. 2021 Jun 10;31(8):847–60. doi: 10.1038/s41422-021-00519-4 (PMC8190750; doi:10.1038/s41422-021-00519-4)
Supplement: Supplementary file 9 — Supplementary information, Fig. S9 [file 41422_2021_519_MOESM9_ESM.pdf]

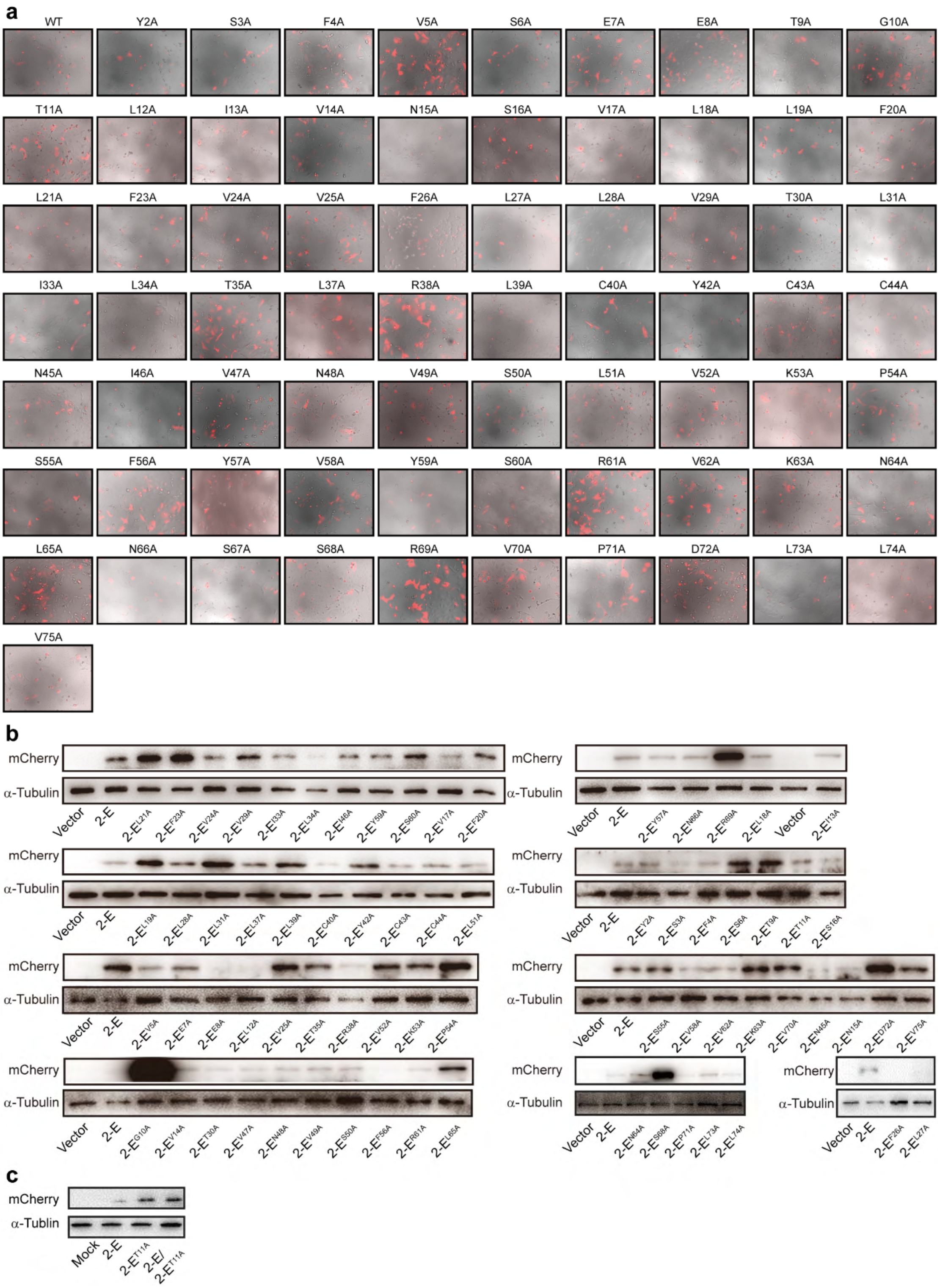

Supplementary information, Fig. S9: Expression level of each mutation after transfection.

a Images for each mutation with mCherry tag. **b** Expression level of 2-E mutant in Vero E6 cells lines. **c** Expression level for Vero E6 cells after transfecting plasmids as indicated.
