# Supplementary material for: SARS-CoV-2 envelope protein causes acute respiratory distress syndrome (ARDS)-like pathological damages and constitutes an antiviral target
Source: Cell Res. 2021 Jun 10;31(8):847–60. doi: 10.1038/s41422-021-00519-4 (PMC8190750; doi:10.1038/s41422-021-00519-4)
Supplement: Supplementary file 10 — Supplementary information, Fig. S10 [file 41422_2021_519_MOESM10_ESM.pdf]

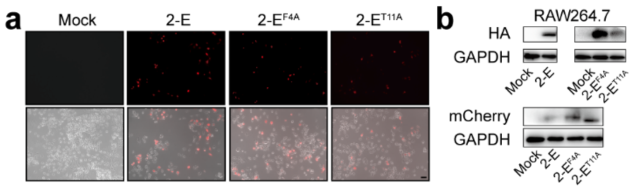

Supplementary information, Fig. S10: Expression level of 2-E in transfected RAW264.7 cells.

a The fluorescence detection of 2-E-mCherry transfected RAW264.7 cells (bar, 50 μm).

b The expression level of 2-E in 2-E-HA (Up) or 2-E-mCherry (Down) transfected RAW264.7 cells.
