# Supplementary material for: SARS-CoV-2 envelope protein causes acute respiratory distress syndrome (ARDS)-like pathological damages and constitutes an antiviral target
Source: Cell Res. 2021 Jun 10;31(8):847–60. doi: 10.1038/s41422-021-00519-4 (PMC8190750; doi:10.1038/s41422-021-00519-4)
Supplement: Supplementary file 11 — Supplementary information, Fig. S11 [file 41422_2021_519_MOESM11_ESM.pdf]

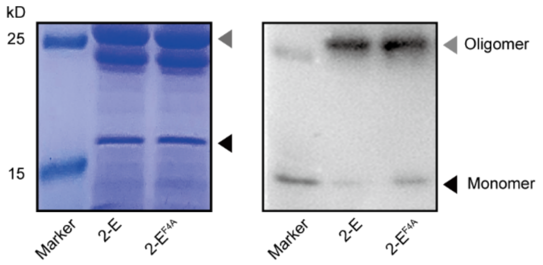

Supplementary information, Fig. S11: Purification of 2-E and 2-E^{F4A} proteins. Purification of full-length 2-E and 2-E^{F4A} proteins with Ni-NTA affinity chromatography. Left: 15% SDS-PAGE gel with coomassie blue staining; Right: Western blot probed with anti-his antibody.
