# Supplementary material for: SARS-CoV-2 envelope protein causes acute respiratory distress syndrome (ARDS)-like pathological damages and constitutes an antiviral target
Source: Cell Res. 2021 Jun 10;31(8):847–60. doi: 10.1038/s41422-021-00519-4 (PMC8190750; doi:10.1038/s41422-021-00519-4)
Supplement: Supplementary file 12 — Supplementary information, Fig. S12 [file 41422_2021_519_MOESM12_ESM.pdf]

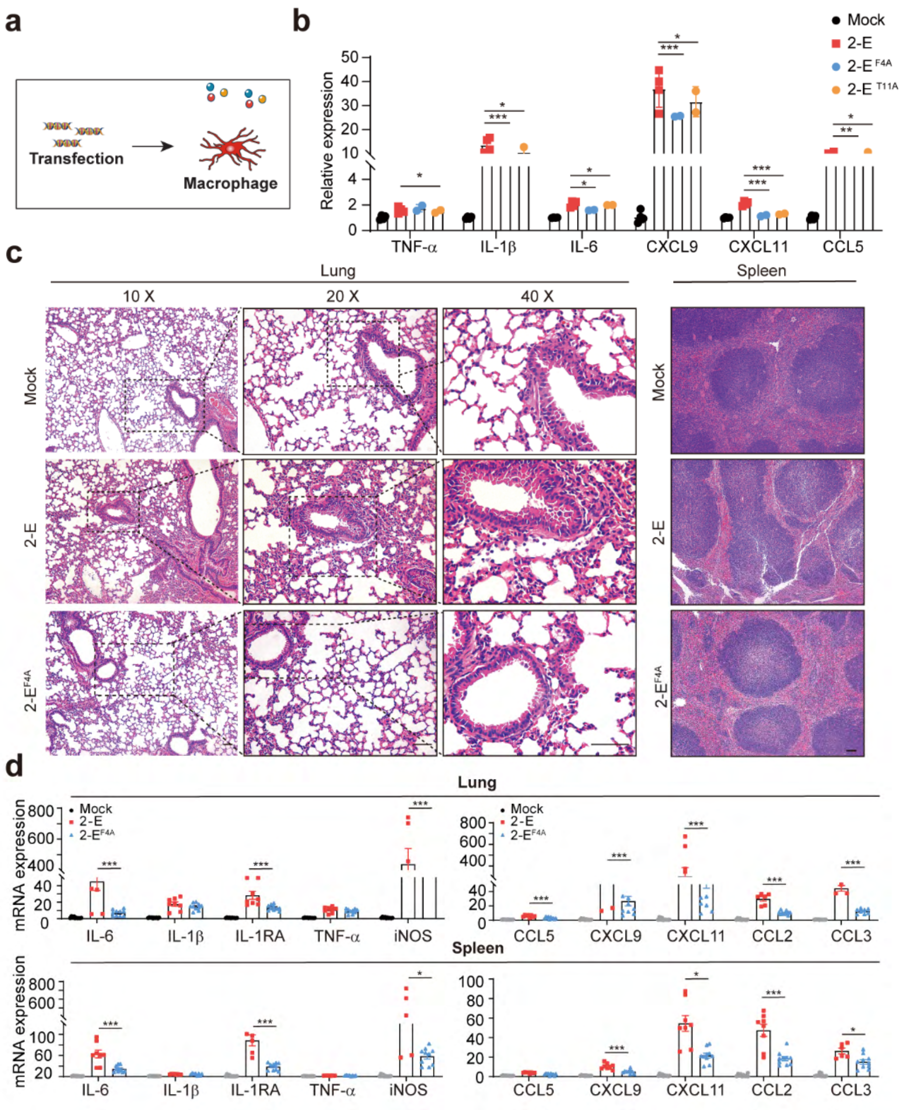

Supplementary information, Fig. S12: 2-E mutant channels relieve robust immune responses in vitro and in vivo.

a Schematic for detecting the immune response of macrophages. **b** Expression of cytokines and chemokines following transfection of 2-E,2-E^{F4A} and 2-E^{T11A} plasmids, measuring mRNA expression via qRT-PCR. **c** Histopathology of lungs and spleens from 2-E and 2-E^{F4A} protein treatment groups (bar, 10 μm). **d** qRT-PCR analysis of cytokine levels after 6 h treatment (n>4). All data are representative of three independent experiments. **P < 0.05; **P < 0.01; ***P < 0.001;* unpaired Student's t test. All error bars are SEM.
