# Supplementary material for: SARS-CoV-2 envelope protein causes acute respiratory distress syndrome (ARDS)-like pathological damages and constitutes an antiviral target
Source: Cell Res. 2021 Jun 10;31(8):847–60. doi: 10.1038/s41422-021-00519-4 (PMC8190750; doi:10.1038/s41422-021-00519-4)
Supplement: Supplementary file 13 — Supplementary information, Fig. S13 [file 41422_2021_519_MOESM13_ESM.pdf]

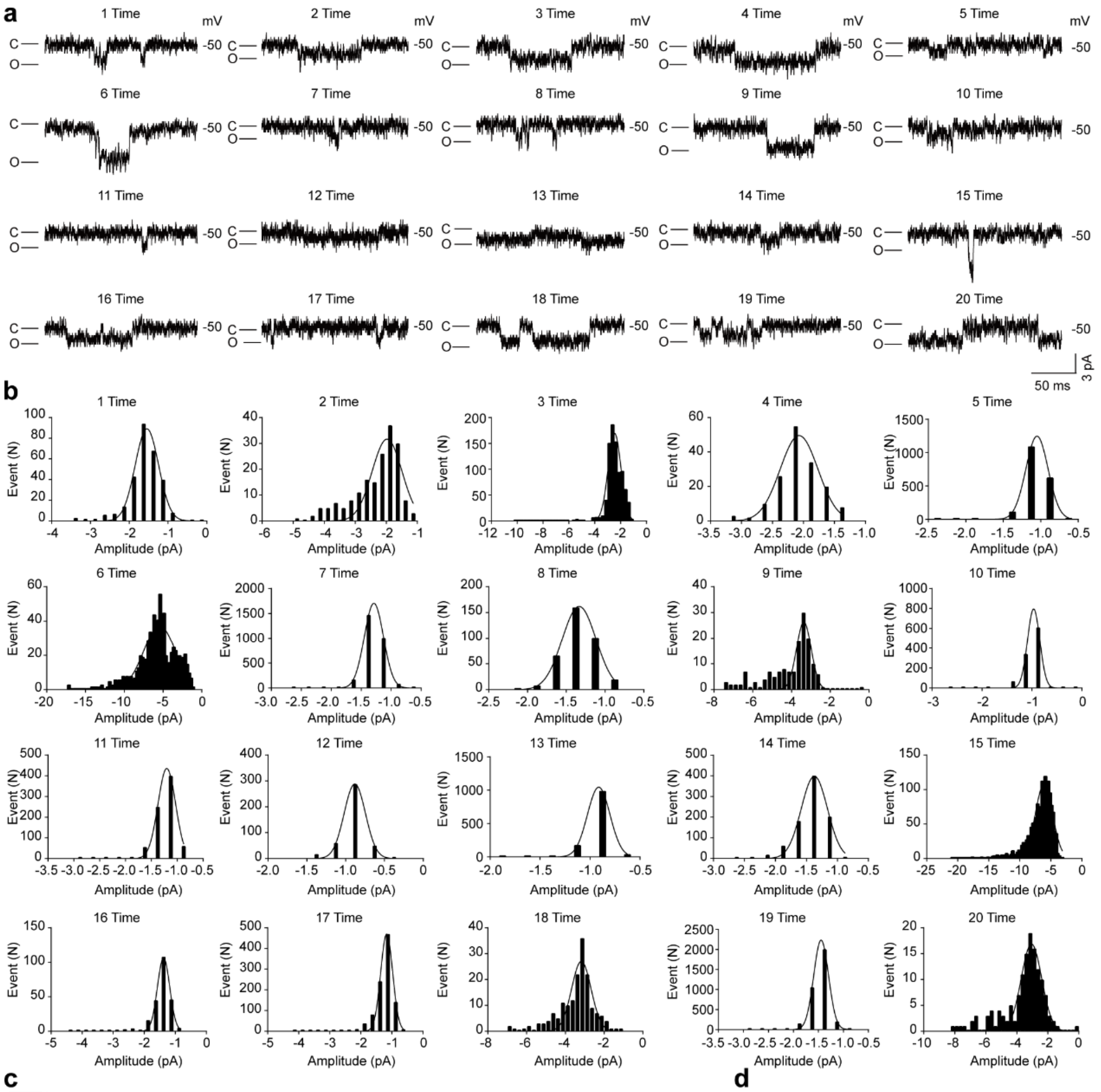

c

Time	Amplitude (pA)	Time	Amplitude (pA)	Time	Amplitude (pA)	Time	Amplitude (pA)	Time	Amplitude (pA)
1	-1.543	2	-1.973	3	-2.474	4	-2.070	5	-1.049
6	-5.444	7	-1.288	8	-1.334	9	-3.362	10	-0.965
11	-1.201	12	-0.882	13	-0.913	14	-1.372	15	-6.104
16	-1.377	17	-1.165	18	-3.177	19	-1.445	20	-3.055

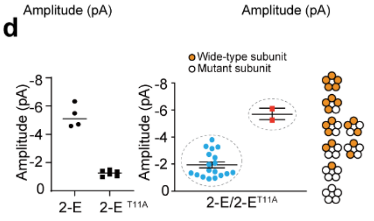

Supplementary information, Fig. S13: Dominant negative mutation Thr11Ala (2-ET11A) impairs 2-E channel activity.

a Representative trace for 2-E/2-E^{T11A} mixture proteins in lipid bilayers with PC/PS = 3:2 lipids at -50 mV. The protein was reconstituted in asymmetric 50:500 mM KCl solutions (*trans* : *cis*) . **b** Current amplitude of each 2-E/2-E^{T11A} trace by Gaussian

fitting. c, Statistics of each 2-E/2-E^{T11A} currents. d, Current amplitude of 2-E or 2-E^{T11A} (Left) and mixture of 2-E/2-E^{T11A} (Right).
