# Supplementary material for: SARS-CoV-2 envelope protein causes acute respiratory distress syndrome (ARDS)-like pathological damages and constitutes an antiviral target
Source: Cell Res. 2021 Jun 10;31(8):847–60. doi: 10.1038/s41422-021-00519-4 (PMC8190750; doi:10.1038/s41422-021-00519-4)
Supplement: Supplementary file 14 — Supplementary information, Fig. S14 [file 41422_2021_519_MOESM14_ESM.pdf]

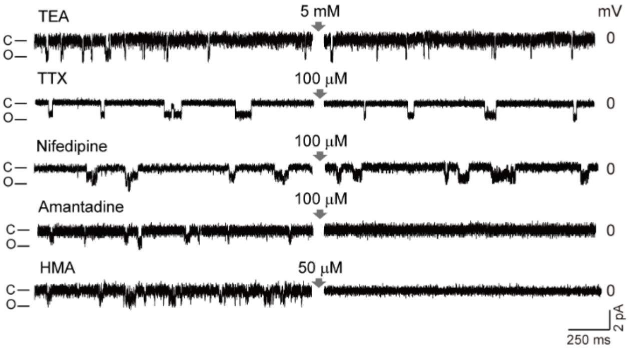

Supplementary information, Fig. S14: Representative single-channel traces after exposure to the indicated compounds. Representative single-channel traces after exposure to the indicated compounds at indicated concentrations. Once ion channel conductance was detected, compounds were added to the *trans* chamber while stirring to facilitate binding of the compound to the channel. The gray arrow indicates the application of compounds (n $\geqslant$ 3).
