# Supplementary material for: SARS-CoV-2 envelope protein causes acute respiratory distress syndrome (ARDS)-like pathological damages and constitutes an antiviral target
Source: Cell Res. 2021 Jun 10;31(8):847–60. doi: 10.1038/s41422-021-00519-4 (PMC8190750; doi:10.1038/s41422-021-00519-4)
Supplement: Supplementary file 15 — Supplementary information, Fig. S15 [file 41422_2021_519_MOESM15_ESM.pdf]

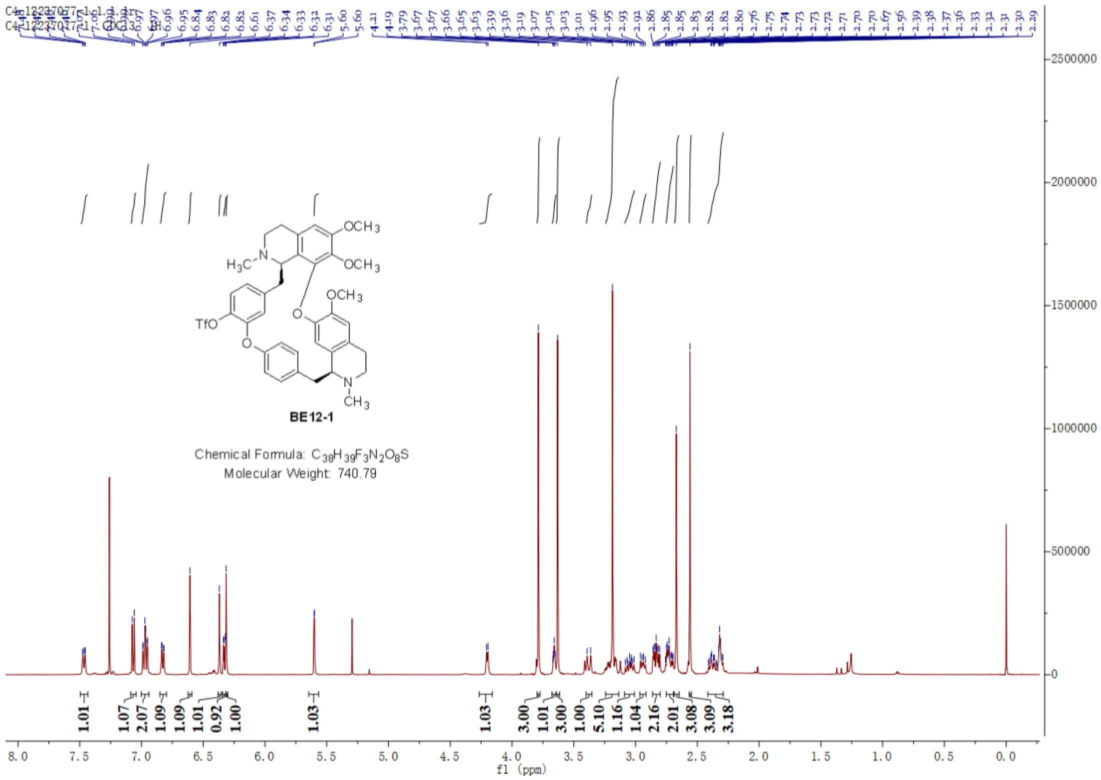

^{1}H NMR spectrum of BE-12-1

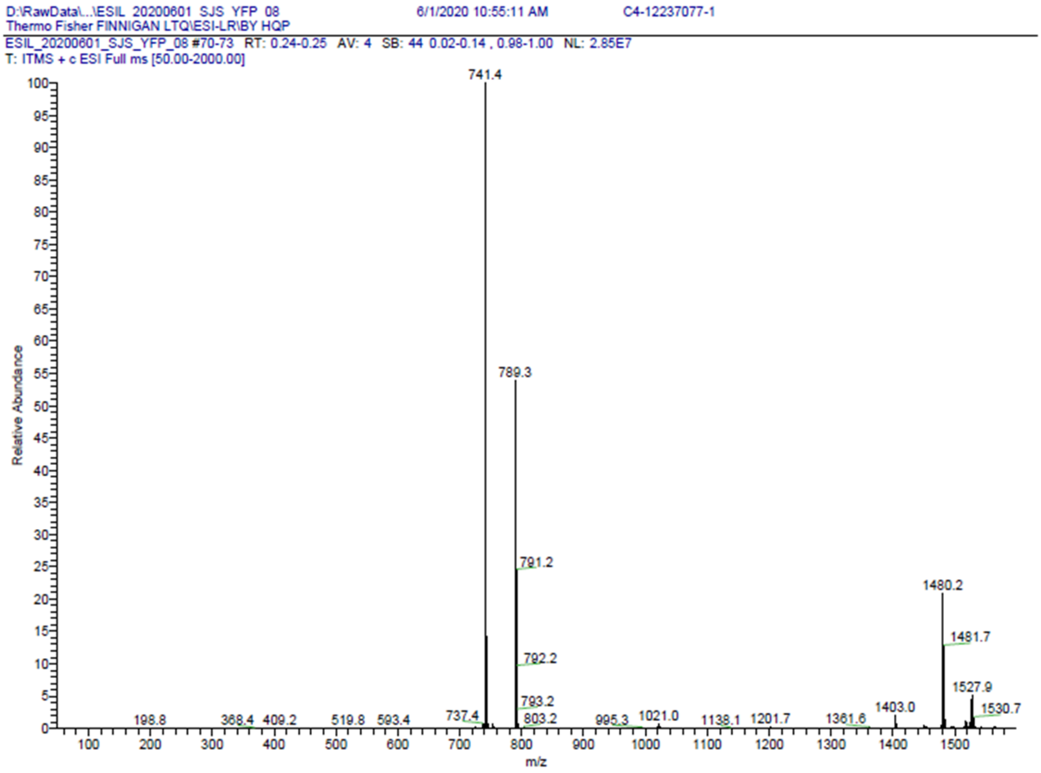

MS spectrum of BE-12-1

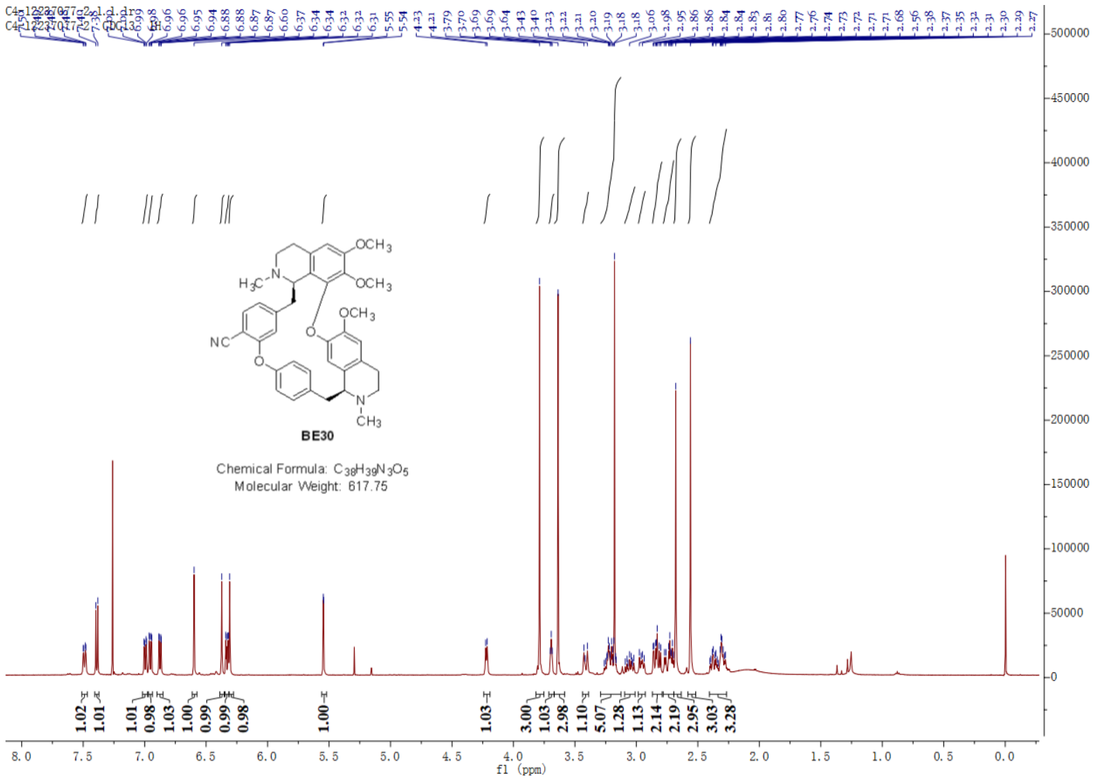

^{1}H NMR spectrum of BE-30

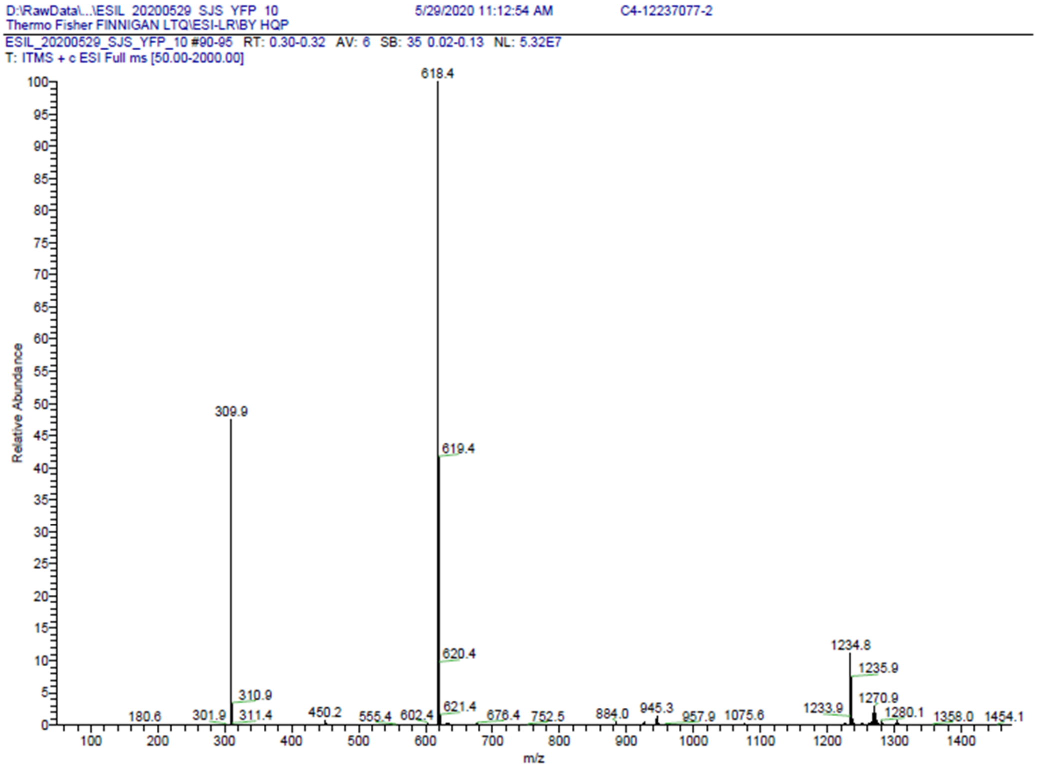

MS spectrum of BE-30

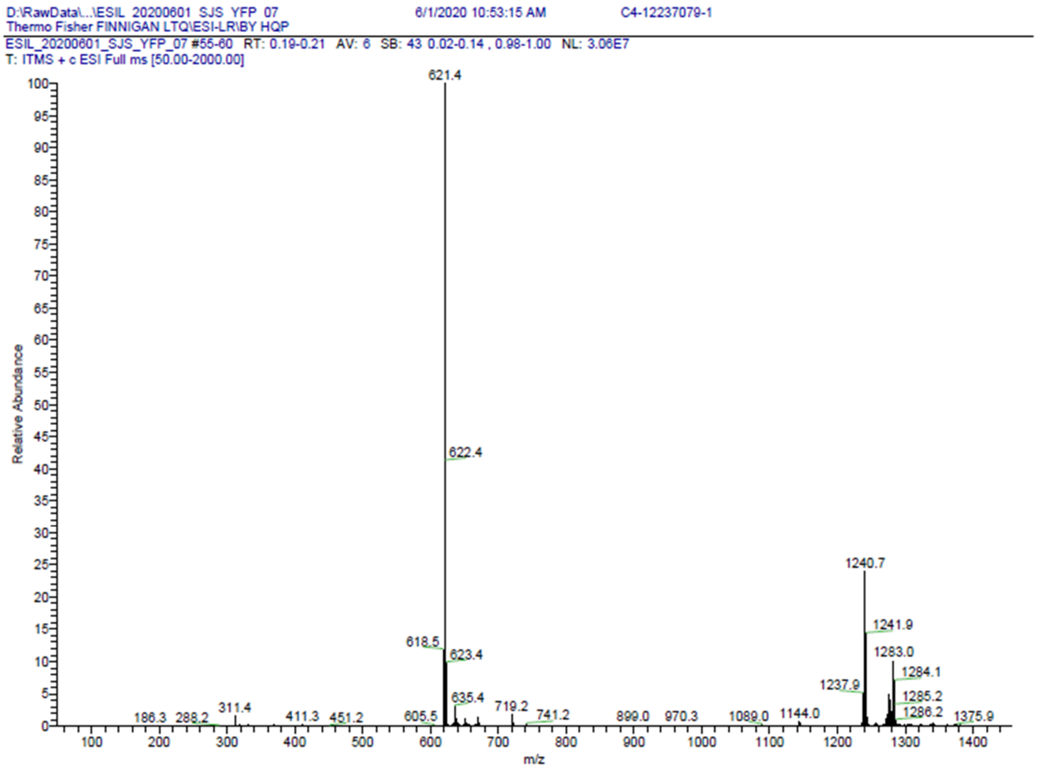

MS spectrum of BE-12-2

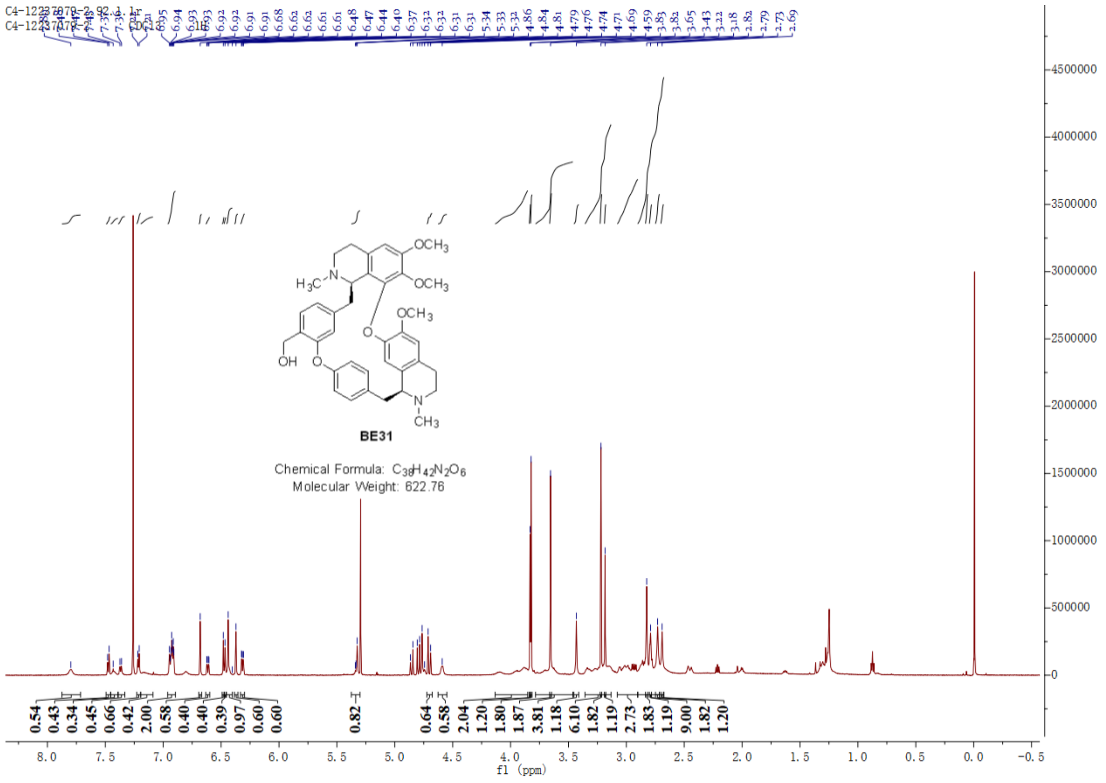

^{1}H NMR spectrum of BE-31

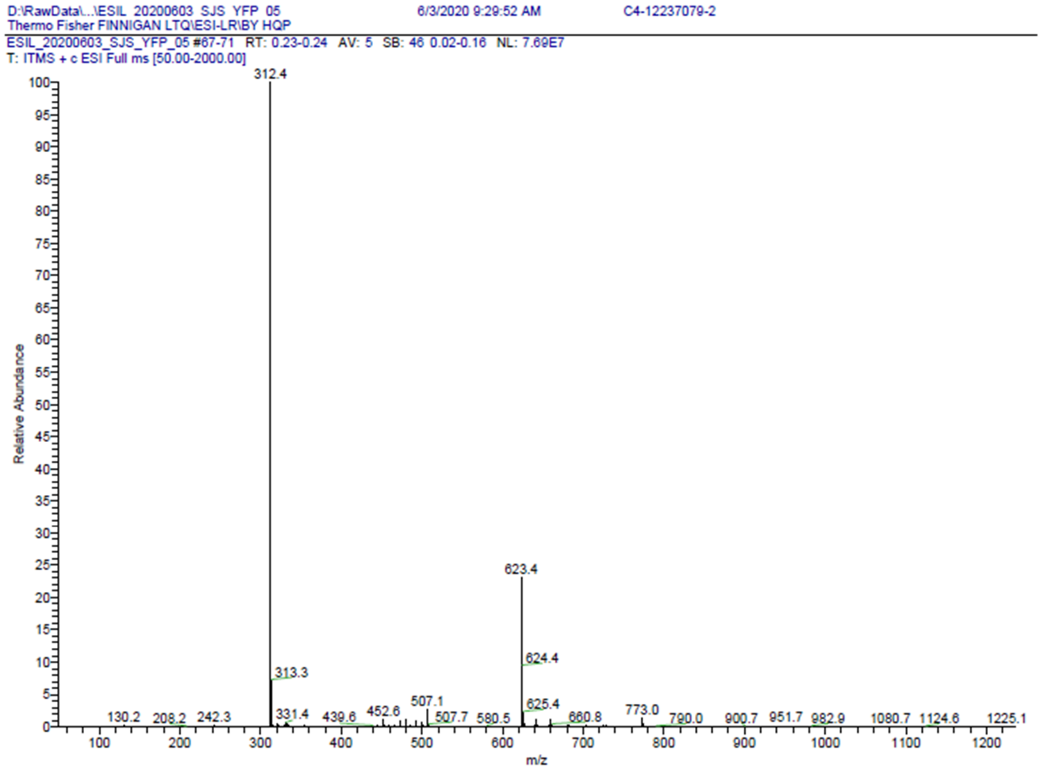

MS spectrum of BE-31

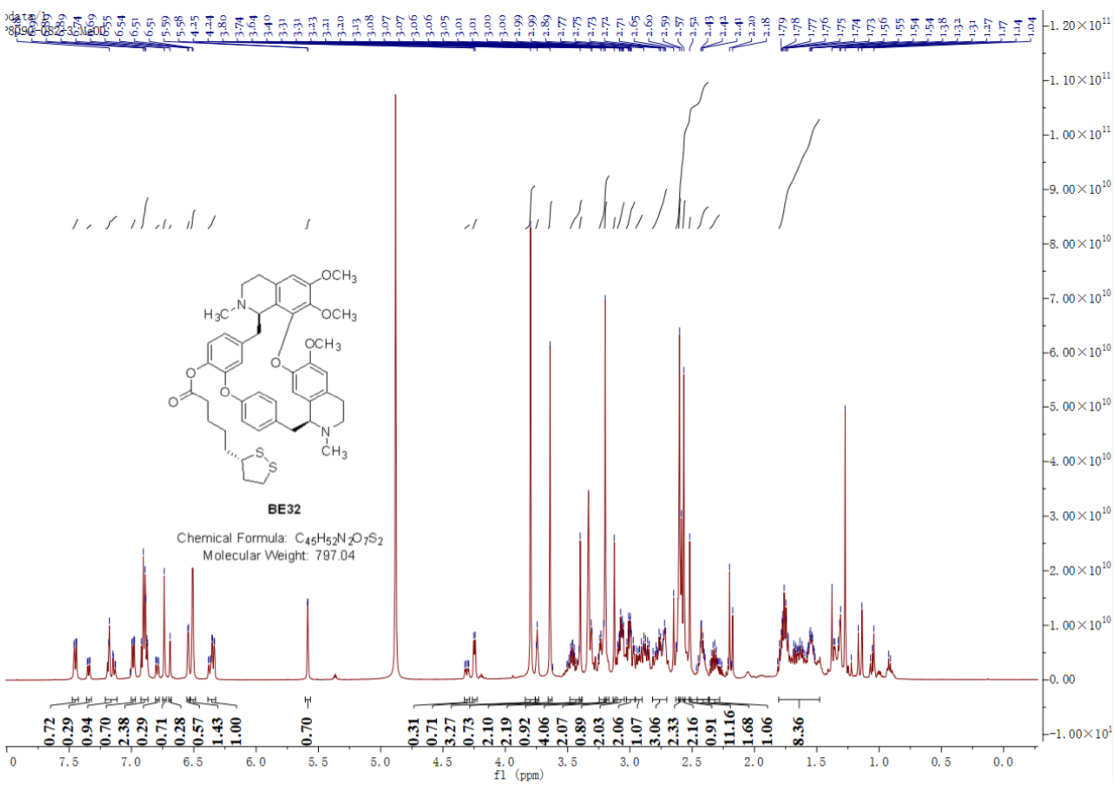

^{1}H NMR spectrum of BE-32

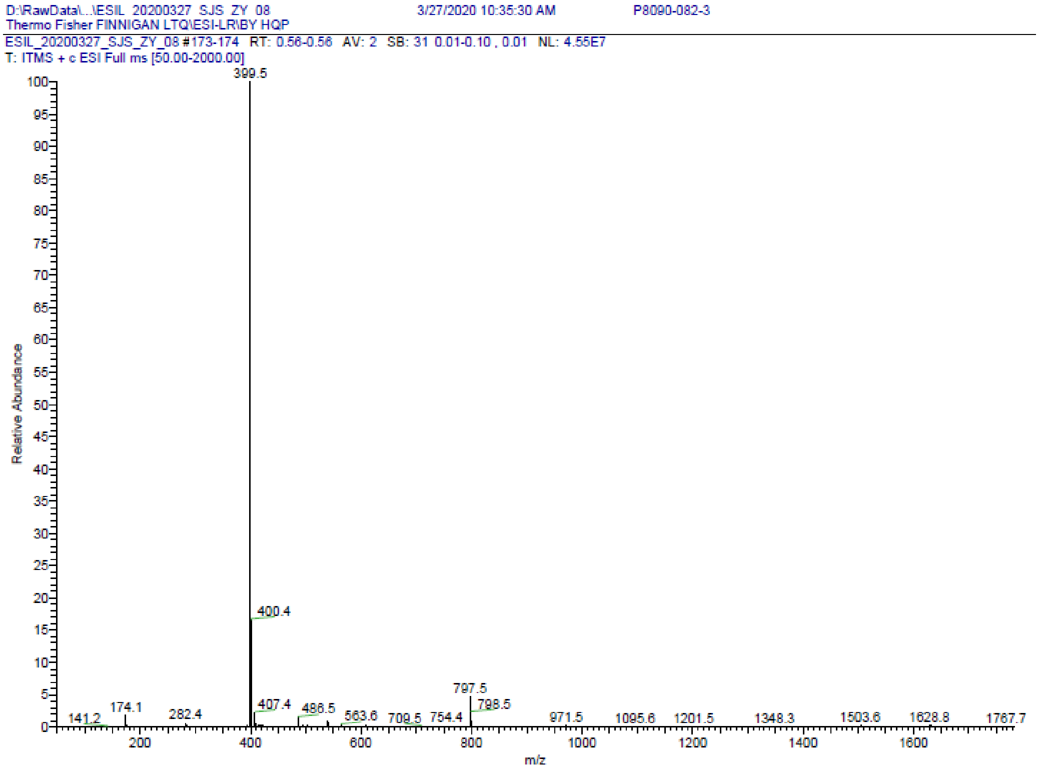

MS spectrum of BE-32

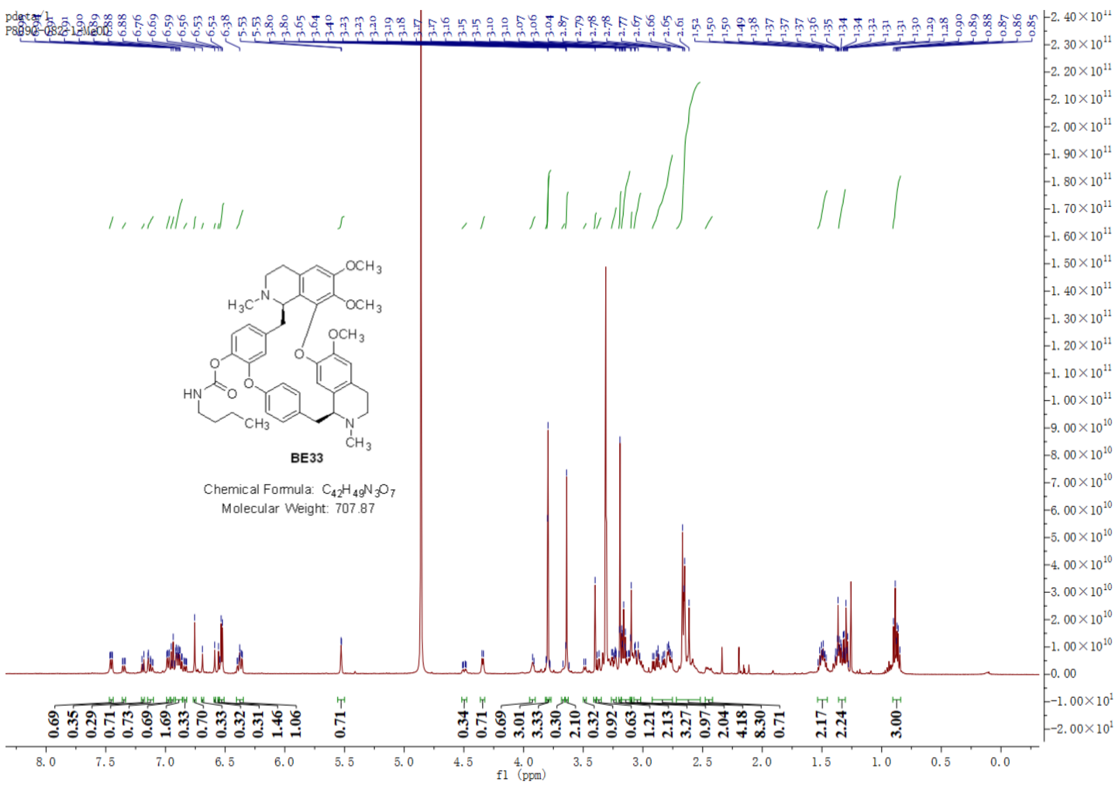

^{1}H NMR spectrum of BE-33

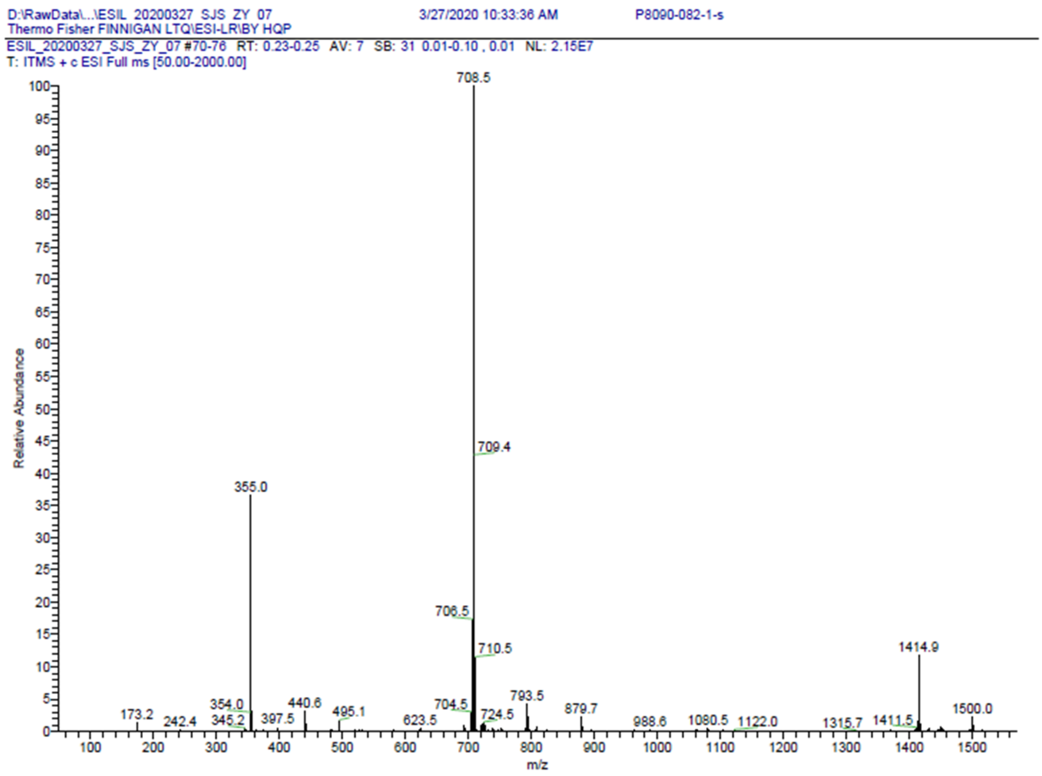

MS spectrum of BE-33

Supplementary information, Fig. S15: Spectral data for compounds BE-12-1, BE-30, BE-31, BE-32 and BE-33.
