# Supplementary material for: SARS-CoV-2 envelope protein causes acute respiratory distress syndrome (ARDS)-like pathological damages and constitutes an antiviral target
Source: Cell Res. 2021 Jun 10;31(8):847–60. doi: 10.1038/s41422-021-00519-4 (PMC8190750; doi:10.1038/s41422-021-00519-4)
Supplement: Supplementary file 16 — Supplementary information, Fig. S16 [file 41422_2021_519_MOESM16_ESM.pdf]

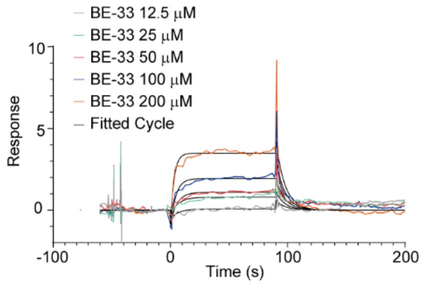

Ka (1/Ms)	Kd (1/s)	KD(M)
976.1	0.1317	1.349E-4

Supplementary information, Fig. S16: Direct binding of BE-33 with 2-E channels.

SPR analysis of BE-33 binding to 2-E protein. The amount of the ligand bound to 2-E was subsequently detected, and association and dissociation curves were obtained (Up). Dissociation constant (KD) was calculated after fitting these data to fit curves through non-linear regression analysis (Down).
