# Supplementary material for: SARS-CoV-2 envelope protein causes acute respiratory distress syndrome (ARDS)-like pathological damages and constitutes an antiviral target
Source: Cell Res. 2021 Jun 10;31(8):847–60. doi: 10.1038/s41422-021-00519-4 (PMC8190750; doi:10.1038/s41422-021-00519-4)
Supplement: Supplementary file 17 — Supplementary Methods [file 41422_2021_519_MOESM17_ESM.pdf]

Supplementary information, Method:

Inhibitor synthesis

General procedure. Reagents and solvents were purchased from commercial sources and used without purification. HSGF 254 (0.15−0.2 mm thickness) was used for analytical thin-layer chromatography (TLC). All products were characterized by their NMR and MS spectra. ^{1}H NMR spectra was recorded on a Bruker spectrometer with TMS as the internal standard. Chemical shifts were given in δ values (ppm) and coupling constants (*J*) were given in Hz. Signal multiplicities were characterized as s (singlet), d (doublet), t (triplet), m (multiplet) and br (broad). The mass spectra were determined on a Thermo-Fisher Finnigan LTQ mass spectrometer. HPLC spectra were recorded by an Agilent 1100 (Agilent Corporation) with SunFire C18 (150 × 4.6 mm, 5 μm) with two solvent systems (acetonitrile/0.02 M 1-octanesulfonic acid sodium in water). Purity was determined by reversed-phase HPLC and was ⩾ 95% for **BE-30**, **BE-31**, **BE-32** and **BE-33**.

Scheme S1: Synthetic route of compounds BE-30 and BE-31[*a*]

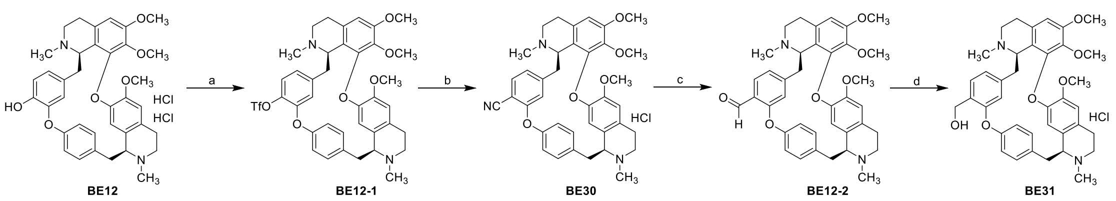

[a]Reagents and conditions: (a) Tf_2O, Et_3N, DMAP, DCM, 0°C–rt; (b) $Zn(CN)_2$, $Pd_2(dba)_3$, DPPF, zinc dust, DMAC, 150°C; (c) Raney nickel, formic acid, 90°C; (d) $NaBH_4$, MeOH, 0°C–rt.

Synthesis of BE-12-1

To a 10 mL round-bottom flask was added phenol **BE-12** (185 mg, 0.271 mM), DCM (2 mL), DMAP (16.6 mg, 0.136 mM, 0.5 eq) and triethylamine (0.189 mL, 1.355 mM, 5 eq). The clear solution was stirred and cooled in an ice bath for 10 min. Triflic anhydride (0.091 mL, 0.542 mM, 2 eq) was added dropwise over 2 mins. The reaction was slowly warmed to room temperature. After 30 mins, the reaction was diluted with DCM (5 mL) and quenched with 0.5 M aqueous HCl (1.1 mL). The organic layer was separated and washed with sat. aq. $NaHCO_3$ (5 mL), then brine (5 mL), dried over Na_2SO_4, filtered, and concentrated via rotary evaporation. The crude product was purified by silica gel chromatography (DCM:MeOH = 80:1) to give triflate **BE-12-1** as a light-yellow solid (160 mg, 0.217 mM, 80% yield). ^1H NMR (500 MHz, Chloroform-

d) δ 7.47 (dd, *J* = 8.5, 2.2 Hz, 1H), 7.07 (d, *J* = 8.3 Hz, 1H), 6.97 (td, *J* = 8.8, 2.4 Hz, 2H), 6.83 (dd, *J* = 8.4, 2.0 Hz, 1H), 6.61 (s, 1H), 6.37 (s, 1H), 6.34–6.32 (m, 1H), 6.31 (s, 1H), 5.60 (d, *J* = 2.0 Hz, 1H), 4.20 (d, *J* = 5.9 Hz, 1H), 3.79 (s, 3H), 3.66 (d, *J* = 3.4 Hz, 1H), 3.63 (s, 3H), 3.38 (d, *J* = 14.4 Hz, 1H), 3.24–3.14 (m, 5H), 3.05 (m, 1H), 2.94 (m, 1H), 2.83 (m, 2H), 2.73 (m, 2H), 2.67 (s, 3H), 2.56 (s, 3H), 2.41–2.29 (m, 3H). ESI-MS (*m/z*): 741.4 $[M + H]^+$.

Synthesis of BE-30

A mixture of triflate **BE-12-1** (170 mg, 0.230 mM), $Zn(CN)_2$ (40 mg, 0.345 mM, 1.5 eq), $Pd_2(dba)_3$ (11 mg, 0.012 mM, 0.05 eq), 1,1'-bis(diphenylphosphino)ferrocene (13 mg, 0.023 mM, 0.1 eq) and zinc dust (2 mg, 0.03 mM, 0.13 eq) in DMAC (2 mL) in a 10 mL round-bottom flask was purged with nitrogen for three times at room temperature. Then the mixture was heated to 150 °C and stirred at this temperature for 3 h. After cooling to room temperature, ethyl acetate (10 mL) was added and the resulting mixture was filtered. The filtrate was washed with water (2 × 5 mL), brine (5 mL), dried over Na_2SO_4, filtered, and concentrated via rotary evaporation. The crude product was purified by silica gel chromatography (DCM:MeOH = 80:1) to give free

base of **BE-30** as a light-yellow solid (110 mg, 0.178 mM, 78% yield). ^{1}H NMR (500 MHz, Chloroform-*d*) δ 7.49 (dd, *J* = 8.4, 2.3 Hz, 1H), 7.39 (d, *J* = 7.9 Hz, 1H), 6.99 (dd, *J* = 8.2, 2.3 Hz, 1H), 6.95 (dd, *J* = 8.3, 2.6 Hz, 1H), 6.87 (dd, *J* = 7.9, 1.4 Hz, 1H), 6.60 (s, 1H), 6.37 (s, 1H), 6.33 (dd, *J* = 8.3, 2.6 Hz, 1H), 6.31 (s, 1H), 5.55 (d, *J* = 1.4 Hz, 1H), 4.22 (d, *J* = 6.0 Hz, 1H), 3.79 (s, 3H), 3.69 (d, *J* = 3.8 Hz, 1H), 3.64 (s, 3H), 3.44−3.39 (m, 1H), 3.28−3.14 (m, 5H), 3.06 (m, 1H), 2.96 (m, 1H), 2.88−2.79 (m, 2H), 2.78−2.70 (m, 2H), 2.68 (s, 3H), 2.56 (s, 3H), 2.40−2.27 (m, 3H). To a solution of free base of **BE-30** (105 mg) in methanol (1 mL) was added concentrated hydrochloric acid (14.9 μL). The resulting solution was stirred at room temperature for 5 min and then concentrated via rotary evaporation. Methyl tert-butyl ether (1 mL) was added to the residue and the precipitation was filtered and dried under vacuum at 45 °C to provide hydrochloride salt of **BE-30** as a yellow solid (90 mg). ESI-MS (*m/z*): 618.4 $[M + H]^{+}$.

Synthesis of BE-12-2

A mixture of hydrochloride salt of **BE-30** (75 mg, 0.115 mM) and Raney nickel (100 mg) was suspended in formic acid (3 mL) and water (0.1 mL) and heated to 90 °C for 2 h. The mixture was then filtered, rinsed with methanol, and concentrated. To the

resulting residue was added DCM (5 mL) followed by sat. aq. $NaHCO_3$ until basic. The organic layer was separated, dried over Na_2SO_4, and concentrated. The crude product was purified by silica gel chromatography (DCM:MeOH = 75:1) to give aldehyde **BE-12-2** as a light-yellow oil (38 mg, 0.061 mM, 57% yield). ESI-MS (*m/z*): 621.4 $[M + H]^+$.

Synthesis of BE-31

Sodium borohydride (4 mg, 0.106 mM, 1.8 eq) was added to a solution of **BE-12-2** (36 mg, 0.058 mM) in methanol (1 mL) under an ice bath, and the solution was slowly warmed to room temperature. After 2 h, the reaction was quenched with sat. aq. NH_4Cl (1 mL) and extracted with DCM (2 × 2 mL). The combined organic layer was washed with brine (2 mL), dried over Na_2SO_4, filtered, and concentrated via rotary evaporation. The crude product was purified by silica gel chromatography (DCM:MeOH = 65:1) to give free base of **BE-31** as a light-yellow solid (20 mg, 0.032 mM, 55% yield). Two sets of ^1H NMR data representing two rotamers (~3:2) were observed as indicative of the presence of atropisomers in bisbenzylisoquinoline type alkaloids involving planar chirality. ^1H NMR (600 MHz, Chloroform-*d*, major rotamer) δ 7.80 (brs, 1H), 7.37 (d,

J = 8.4 Hz, 1H), 7.21 (d, *J* = 7.3 Hz, 1H), 6.92 (m, 2H), 6.68 (s, 1H), 6.44 (s, 1H), 6.37 (s, 1H), 6.32 (dd, *J* = 8.1, 2.6 Hz, 1H), 5.32 (s, 1H), 4.78 (m, 2H), 4.59 (m, 1H), 4.14–3.85 (m, 2H), 3.82 (s, 3H), 3.78–3.46 (m, 2H), 3.65 (s, 3H), 3.36–3.13 (m, 3H), 3.22 (s, 3H), 3.08–2.91 (m, 3H), 2.90–2.67 (m, 3H), 2.82 (s, 3H), 2.73 (s, 3H); ^{1}H NMR (600 MHz, Chloroform-*d*, minor rotamer) δ 7.47 (d, *J* = 7.8 Hz, 1H), 7.43 (brs, 1H), 7.16 (brs, 1H), 6.92 (m, 2H), 6.61 (dd, *J* = 8.5, 2.7 Hz, 1H), 6.48 (s, 1H), 6.47 (s, 1H), 6.44 (s, 1H), 5.32 (s, 1H), 4.78 (m, 2H), 4.75 (m, 1H), 4.14–3.85 (m, 2H), 3.83 (s, 3H), 3.78–3.46 (m, 2H), 3.43 (s, 3H), 3.36–3.13 (m, 3H), 3.18 (s, 3H), 3.08–2.91 (m, 3H), 2.90–2.67 (m, 3H), 2.79 (s, 3H), 2.69 (s, 3H). To a solution of free base of **BE-31** (16 mg) in methanol (0.5 mL) was added concentrated hydrochloric acid (2.2 µL). The resulting solution was stirred at room temperature for 5 mins and then concentrated via rotary evaporation. Acetonitrile (0.5 mL) was added to the residue and the precipitation was filtered and dried under vacuum at 45 °C to provide hydrochloride salt of **BE-31** as an off-white solid (13 mg). ESI-MS (*m/z*): 623.4 $[M + H]^+$.

Scheme S2: Synthetic route of compound BE-32*ᵃ*

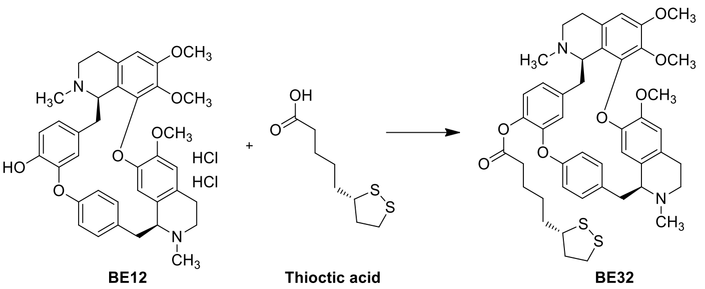

[a]Reagents and conditions: EDCI, DMAP, DCM, rt.

Synthesis of BE-32

EDCI (43 mg, 0.224 mM, 1.5 eq) and DMAP (4 mg, 0.033 mM, 0.2 eq) were added to a suspension of phenol **BE-12** (100 mg, 0.147 mM) and L-thioctic acid (31 mg, 0.150 mM, 1.0 eq) in DCM (3 mL) at room temperature. The mixture was stirred for 5 h before water (2 mL) was added. The organic layer was separated, washed with brine (2 mL), dried over Na_2SO_4, filtered, and concentrated. The crude product was purified by silica gel chromatography (DCM:MeOH = 50:1) to give **BE-32** as a light-yellow solid (63 mg, 0.079 mM, 54% yield). Two sets of ^{1}H NMR data representing two rotamers (~7:3) were observed. ^{1}H NMR (500 MHz, Methanol-d_4, major rotamer) δ 7.45 (dd, J = 8.5, 2.2 Hz, 1H), 7.16 (m, 1H), 6.99 (dd, J = 8.3, 2.2 Hz, 1H), 6.90 (m, 2H), 6.74 (s, 1H), 6.51 (s, 1H × 2), 6.34 (dd, J = 8.2, 2.6 Hz, 1H), 5.59 (d, J = 1.7 Hz, 1H), 4.25 (d, J = 5.7 Hz, 1H), 3.80 (s, 3H), 3.75 (d, J = 3.7 Hz, 1H), 3.64 (s, 3H), 3.46 (m, 1H),

3.24–3.16 (m, 2H), 3.20 (s, 3H), 3.10–3.04 (m, 2H), 3.03–2.96 (m, 2H), 2.93 (m, 1H), 2.82–2.70 (m, 3H), 2.63–2.37 (m, 5H), 2.60 (s, 3H), 2.57 (s, 3H), 2.32 (m, 1H), 1.80–1.48 (m, 8H); ^{1}H NMR (500 MHz, Methanol-d_4, minor rotamer) δ 7.34 (dd, J = 8.5, 2.2 Hz, 1H), 7.16 (m, 1H), 6.90 (m, 2H), 6.79 (dd, J = 8.4, 2.7 Hz, 1H), 6.69 (s, 1H), 6.55 (s, 1H), 6.54 (s, 1H), 6.37 (m, 1H), 4.31 (dd, J = 11.4, 4.2 Hz, 1H), 3.80 (m, 4H), 3.46 (m, 2H), 3.40 (s, 3H), 3.24–3.16 (m, 2H), 3.13 (s, 3H), 3.10–3.04 (m, 2H), 3.03–2.96 (m, 2H), 2.93 (m, 1H), 2.82–2.70 (m, 3H), 2.63–2.37 (m, 5H), 2.59 (s, 3H), 2.52 (s, 3H), 2.32 (m, 1H), 1.80–1.48 (m, 8H). ESI-MS (*m/z*): 797.5 $[M + H]^+$.

Scheme S3: Synthetic route of compound BE-33[a]

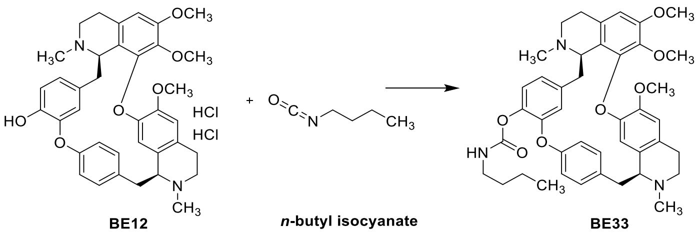

[a]Reagents and conditions: Et_3N, CH_3CN, rt.

Synthesis of BE-33

Triethylamine (0.073 mL, 0.524 mM, 3.6 eq) was added to a suspension of phenol **BE-12** (100 mg, 0.147 mM) and *n*-butyl isocyanate (22 mg, 0.222 mM, 1.5 eq) in acetonitrile (3 mL) at room temperature. The mixture was stirred for 2 h, concentrated and subjected to silica gel chromatography (DCM:MeOH=30:1) to give **BE-33** as a light-yellow solid (72 mg, 0.102 mM, 69% yield). Two sets of ^{1}H NMR data representing two rotamers (~7:3) were observed. ^{1}H NMR (500 MHz, Methanol-d_4, major rotamer) δ 7.45 (dd, *J* = 8.4, 2.3 Hz, 1H), 7.13 (m, 1H), 6.98 (dd, *J* = 7.9, 2.1 Hz, 1H), 6.94 (d, *J* = 8.1 Hz, 1H), 6.89 (m, 1H), 6.76 (s, 1H), 6.53 (s, 1H), 6.52 (s, 1H), 6.37 (dd, *J* = 8.2, 2.6 Hz, 1H), 5.53 (d, *J* = 1.9 Hz, 1H), 4.34 (d, *J* = 5.9 Hz, 1H), 3.92 (m, 1H), 3.80 (s, 3H), 3.64 (s, 3H), 3.37 (m, 1H), 3.24 (m, 1H), 3.19 (s, 3H), 3.17 (m, 3H), 3.06 (m, 2H), 2.92–2.76 (m, 4H), 2.72–2.42 (m, 3H), 2.67 (s, 3H), 2.65 (s, 3H), 1.49 (m, 2H), 1.33 (m, 2H), 0.89 (t, *J* = 7.3 Hz, 3H); ^{1}H NMR (500 MHz, Methanol-d_4, minor rotamer) δ 7.35 (dd, *J* = 8.5, 2.3 Hz, 1H), 7.19 (d, *J* = 8.2 Hz, 1H), 6.89 (m, 2H), 6.83 (dd, *J* = 8.4, 2.7 Hz, 1H), 6.69 (s, 1H), 6.59 (s, 1H), 6.56 (s, 1H), 6.39 (m, 1H), 4.50 (dd, *J* = 11.5, 4.2 Hz, 1H), 3.80 (m, 4H), 3.67 (m, 1H), 3.49 (d, *J* = 7.3 Hz, 1H), 3.40 (s, 3H), 3.24 (m, 1H), 3.17 (m, 3H), 3.10 (s, 3H), 3.06 (m, 2H), 2.92–2.76 (m, 4H),

2.72–2.42 (m, 3H), 2.66 (s, 3H), 2.61 (s, 3H), 1.49 (m, 2H), 1.33 (m, 2H), 0.86 (t, *J* = 7.3 Hz, 3H). ESI-MS (*m/z*): 708.5 $[M + H]^+$.
