# Supplementary material for: SARS-CoV-2 envelope protein causes acute respiratory distress syndrome (ARDS)-like pathological damages and constitutes an antiviral target
Source: Cell Res. 2021 Jun 10;31(8):847–60. doi: 10.1038/s41422-021-00519-4 (PMC8190750; doi:10.1038/s41422-021-00519-4)
Supplement: Supplementary file 18 — Supplementary information, Table S1 [file 41422_2021_519_MOESM18_ESM.pdf]

Supplementary information, Table S1: List of Quantitative Real-time PCR (qRT-PCR) primers.

Primer Name	FWD sequence	REV sequence
CCL12	GCTACCACCATCAGTCCTCA	GGGTCAGCACAGATCTCCTT
CXCL9	GAACGGAGATCAAACCTGCC	CGACGACTTTGGGGTGTTTT
CCL2	AGGTCCCTGTCATGCTTCTG	TCTGGACCCATTCCTTCTTG
CCL3	ATGAAGGTCTCCACCACTGC	CCCAGGTCTCTTTGGAGTCA
CCL5	CCCTCACCATCATCCTCACT	CCTTCGAGTGACAAACACGA
IL-10	ATAACTGCACCCACTTCCCA	GGGCATCACTTCTACCAGGT
IL-6	AGTTGCCTTCTTGGGACTGA	TCCACGATTTCCCAGAGAAC
IL-1RA	CCAGCTCATTGCTGGGTACT	TTCTCAGAGCGGATGAAGGT
IL-2	CCCACTTCAAGCTCCACTTC	ATCCTGGGGAGTTTCAGGTT
IL-8	CGTCCCTGTGACACTCAAGA	TAATTGGGCCAACAGTAGCC
IL-3	GCCAGGGGTCTTCATTCGAG	TTCCACGGTTCCACGGTTAG
IL-1β	GAAGTTGACGGACCCCAAAA	CCACAGCCACAATGAGTGATAC
IL-12	CCGAAACCTGCTGAAGACCA	TGGTTTGGTCCCGTGTGATG
Caspase1	ACTGACTGGGACCCTCAAGT	GCAAGACGTGTACGAGTGGT
Caspase3	CTCTGGTACGGATGTGGACG	CCCCTTCATCACCATGGCTT
Caspase9	ACAGATGGATGCTCCGTGTC	CAAGGTCCTGCCTTGAGAGG
INOS	GTTCTCAGGCCAACAATACAAGA	GTGGACGGGTCGATGTCAC
IFN-β	ACCTACAGGGCGGACTTCAA	GTCTCATTCCACCCAGTGCT

HIF-α	TCAGCATACAGTGGCACTCA	AAGGGAGCCATCATGTTCCA
IP10	AAGTGCTGCCGTCATTTTCT	GTGGCAATGATCTCAACACG
IFN-γ	TCCTGCACCAACATTTCTGA	TACGAGGACGGAGAGCTGTT
TNF-α	TCGTAGCAAACCACCAAGTG	GGAGTAGACAAGGTACAACCCA
COX2	AGAAGGAAATGGCTGCAGAA	GCTCGGCTTCCAGTATTGAG
SARS-2-E	TCGTTTCGGAAGAGACAGGT	CACGAGAGTAAACGTAAAAAGAAGG
GAPDH	AACTTTGGCATTGTGGAAGG	ACACATTGGGGGTAGGAACA
